# Amine-Reactive BODIPY Dye: Spectral Properties and Application for Protein Labeling

**DOI:** 10.3390/molecules27227911

**Published:** 2022-11-16

**Authors:** Ksenia V. Ksenofontova, Anastasia A. Kerner, Alexander A. Ksenofontov, Artyom Yu. Shagurin, Pavel S. Bocharov, Michael M. Lukanov, Airat R. Kayumov, Darya E. Zhuravleva, Zalina I. Iskhakova, Evgeniy E. Molchanov, Dmitriy A. Merkushev, Ilya A. Khodov, Yuriy S. Marfin

**Affiliations:** 1 Department of Inorganic Chemistry, Ivanovo State University of Chemistry and Technology, 10, Sheremetevskiy pr., 153000 Ivanovo, Russia; 2 G. A. Krestov Institute of Solution Chemistry of The Russian Academy of Sciences, 1, ul. Akademicheskaya, 153045 Ivanovo, Russia; 3 Laboratoire de Spectroscopie pour les Interactions, la Réactivité et l’Environnement (UMR CNRS 8516), Université de Lille, CEDEX, 59655 Villeneuve d’Ascq, France; 4 Institute of Fundamental Medicine and Biology, Kazan Federal University, 18, ul. Kremlyovskaya, 420008 Kazan, Russia; 5 Department of Fine Organic Synthesis Technology, Ivanovo State University of Chemistry and Technology, 7, Sheremetevskiy pr., 153000 Ivanovo, Russia; 6 Pacific National University, 136, ul. Tihookeanskaya, 680042 Khabarovsk, Russia

**Keywords:** BODIPY, amine-reactive dye, protein, fluorescent labeling, bioconjugation

## Abstract

A boron-dipyrromethene (BODIPY) derivative reactive towards amino groups of proteins (**NHS-Ph-BODIPY**) was synthesized. Spectroscopic and photophysical properties of amine-reactive **NHS-Ph-BODIPY** and its non-reactive precursor (**COOH-Ph-BODIPY**) in a number of organic solvents were investigated. Both fluorescent dyes were characterized by green absorption (521–532 nm) and fluorescence (538–552 nm) and medium molar absorption coefficients (46,500–118,500 M^−1^·cm^−1^) and fluorescence quantum yields (0.32 – 0.73). Solvent polarizability and dipolarity were found to play a crucial role in solvent effects on **COOH-Ph-BODIPY** and **NHS-Ph-BODIPY** absorption and emission bands maxima. Quantum-chemical calculations were used to show why solvent polarizability and dipolarity are important as well as to understand how the nature of the substituent affects spectroscopic properties of the fluorescent dyes. **NHS-Ph-BODIPY** was used for fluorescent labeling of a number of proteins. Conjugation of **NHS-Ph-BODIPY** with bovine serum albumin (BSA) resulted in bathochromic shifts of absorption and emission bands and noticeable fluorescence quenching (about 1.5 times). It was demonstrated that the sensitivity of BSA detection with **NHS-Ph-BODIPY** was up to eight times higher than with Coomassie brilliant blue while the sensitivity of PII-like protein PotN (PotN) detection with **NHS-Ph-BODIPY** and Coomassie brilliant blue was almost the same. On the basis of the molecular docking results, the most probable binding sites of **NHS-Ph-BODIPY** in BSA and PotN and the corresponding binding free energies were estimated.

## 1. Introduction

Detection, tracking, and imaging of proteins are considerable challenges for modern science. One of the ways of tackling this challenge is to label proteins with fluorescent dyes. Fluorescent labels allow imaging various biochemical processes including protein interactions with other biomolecules, protein localization, protein dynamics, enzyme activity, etc. [1,2].

Currently, three main classes of fluorescent labels are widely used [3]: organic dyes, fluorescent proteins, and quantum dots. Each of them has its own advantages and disadvantages. In this regard, the development of new fluorophores that meet all the requirements of bioimaging continues unabated.

Organic dyes being the most popular fluorescent labels include fluoresceins [4], rhodamines [5,6], coumarins [7], dipyrrin complexes [3,8,9,10], pyrenes [11], cyanines [12], etc. The most important feature of organic dyes is the possibility of tuning their fluorescent properties by means of chemical modification and/or environmental change.

Bright members of organic dyes are boron-dipyrromethene (BODIPY) fluorophores and their derivatives [13]. This family of dyes possesses a number of striking properties, such as great stability, high molar absorption coefficients, excellent fluorescence quantum yields, relatively small Stokes shifts, and sharp excitation and emission peaks [14]. Moreover, its ease of chemical modification opens up great possibilities for a synthesis of new fluorescent compounds with predetermined properties.

A rather new promising area in BODIPYs chemistry is the development of dyes reactive towards principal functional groups of proteins [15,16,17,18,19,20,21,22,23,24]. Such fluorescent dyes possess certain reactive substituents capable of covalent binding with proteins, thereby providing their effective labeling. Unfortunately, most of existing reactive BODIPY derivatives have several issues with solubility in water and/or photophysical characteristics. Therefore, the search for reactive fluorescent dyes suitable for conjugation with proteins continues.

In this work, we synthesized a BODIPY derivative reactive towards amino groups of proteins. A comparative analysis of spectroscopic and photophysical properties as well as solvatochromic behavior of the synthesized reactive fluorescent dye and its non-reactive precursor was carried out by means of ultraviolet-visible spectroscopy and steady-state and time-resolved fluorescence spectroscopy supported by quantum chemical calculations. The investigated reactive fluorescent dye was used for labeling of a range of proteins. The sum of the experimental and theoretical results suggests that the synthesized amine-reactive fluorescent dye is a promising fluorescent label for molecular biology and biotechnology.

## 2. Results and Discussion

### 2.1. Synthesis of Reactive BODIPY

**Scheme 1:**
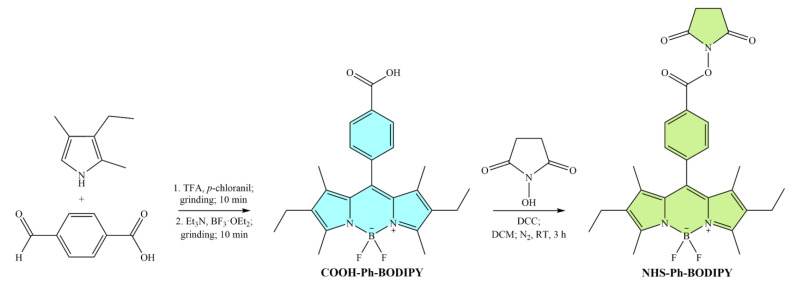
Scheme of reactive BODIPY synthesis.

The amine-reactive fluorescent dye succinimidyl ester of 4-(2,6-diethyl-4,4-difluoro-1,3,5,7-tetramethyl-4-bora-3a,4a-diaza-*s*-indacene-8-yl)-benzoic acid (**NHS-Ph-BODIPY**) was synthesized from 4-(2,6-diethyl-4,4-difluoro-1,3,5,7-tetramethyl-4-bora-3a,4a-diaza-*s*-indacene-8-yl)-benzoic acid (**COOH-Ph-BODIPY**) following the procedure [25] (Scheme 1). Synthetic details and characterization of the compounds obtained are given in Section 3.3.

### 2.2. UV-Vis and Fluorescence Spectroscopy Experiments of BODIPYs

**Figure 1:**
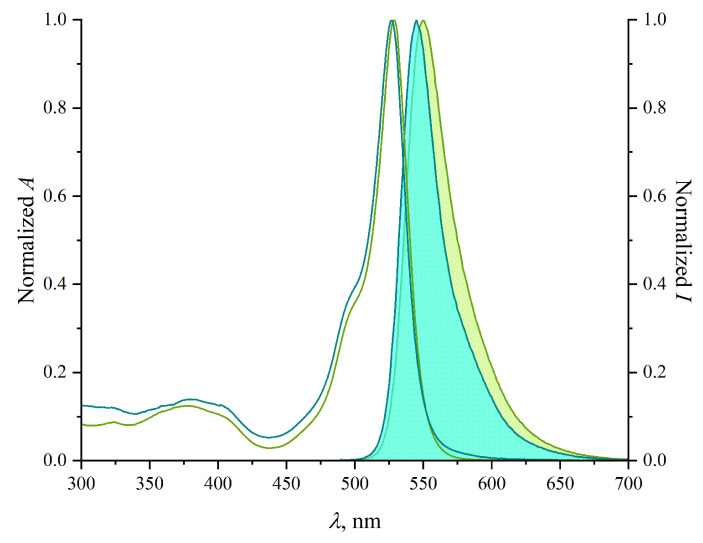
Absorption (unfilled area plot) and emission (filled area plot) spectra of **COOH-Ph-BODIPY** (blue) and **NHS-Ph-BODIPY** (green) in DMSO.

Absorption spectra, emission spectra, fluorescence decay curves as well as a number of photophysical characteristics of synthesized fluorescent dyes **COOH-Ph-BODIPY** and **NHS-Ph-BODIPY** in a range of organic solvents of various nature were obtained (Figure 1 and Figure S1, Table 1).

In Table 1, *λ_abs max_* is the maximum absorption wavelength, *λ_em max_* is the maximum emission wavelength, Δ*ν* is the Stokes shift, and *ε* is the molar absorption coefficient at the maximum absorption wavelength. The asterisk (*) indicates the average fluorescence lifetime $\bar{\tau }$ calculated using Equation (1) [26]:

(1) $$\bar{\tau }=\frac{\alpha_{1}\tau_{1}^{2}+\alpha_{2}\tau_{2}^{2}}{\alpha_{1}\tau_{1}+\alpha_{2}\tau_{2}}$$

where *α* is the amplitude.

The absorption and emission spectra of **COOH-Ph-BODIPY** and **NHS-Ph-BODIPY** are typical for this class of dyes (Figure 1). The absorption spectra gave two bands in the ranges of 521–532 and 375–385 nm due to S_0_–S_1_ and S_0_–S_2_ electron transitions, respectively, while the emission spectra gave one band in the region of 538–552 nm due to S_1_–S_0_ electron transitions (Section 2.3). The absorption and emission bands maxima of **NHS-Ph-BODIPY** are 2–7 nm red shifted compared with the spectra of **COOH-Ph-BODIPY**. The fluorescent dyes are characterized by medium molar absorption coefficients and fluorescence quantum yields which change non-linearly from **COOH-Ph-BODIPY** to **NHS-Ph-BODIPY**. The fluorescence lifetimes of **NHS-Ph-BODIPY** are shorter than those of **COOH-Ph-BODIPY**, while the radiative and non-radiative rate constants change non-linearly.

Solvent effects on spectral characteristics of **COOH-Ph-BODIPY** and **NHS-Ph-BODIPY**, namely the maximum absorption *ν_abs_(max)* and emission *ν_em_(max)* wavenumbers and Stokes shift Δ*ν*, were analyzed in terms of the Kamlet–Taft [27], Catalán [28], and Lippert–Mataga [29] equations.

Table 2 and Table S1 list the estimated regression coefficients *y_0_*, *a* − *d* and their standard errors as well as coefficients of determination R^2^ for the multiple linear regression analyses of the maximum absorption *ν_abs_(max)* and emission *ν_em_(max)* wavenumbers and Stokes shifts Δ*ν* of **COOH-Ph-BODIPY** and **NHS-Ph-BODIPY** according to Equations (5) and (S2) for the solvents chosen.

**Figure 2:**
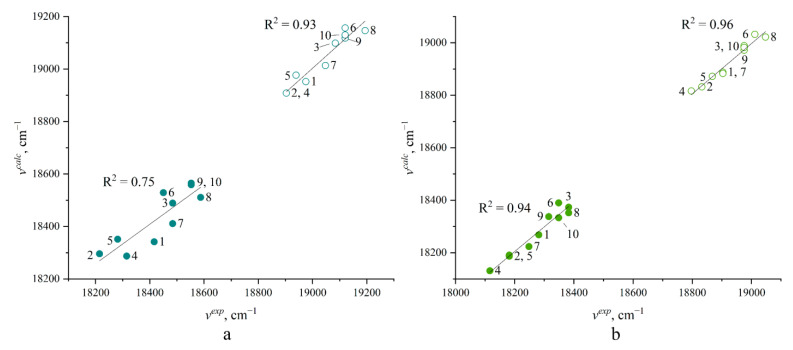
Relationships between experimental and calculated maximum absorption *ν_abs_(max)* (open circle) and emission *ν_em_(max)* (full circle) wavenumbers of **COOH-Ph-BODIPY** (**a**) and **NHS-Ph-BODIPY** (**b**) obtained by multiple linear regression analysis according to Equation (5) in which *y_0_*, *a* − *d* are regression coefficients (numbers refer to organic solvents in Table 1).

The analyses of the *ν_abs_(max)* and *ν_em_(max)* data within the Kamlet–Taft model, in which solvent polarizability and dipolarity effects are combined in the single parameter π*, show poor fits with the R^2^ = 0.44–0.66 and large standard errors on the estimated regression coefficients *a_α_*, *b_β_*, and c_π_* as goodness-of-fit criteria (Table S1). In contrast, the analyses of the same data within the Catalán model, in which solvent polarizability and dipolarity effects are separated by the two parameters *SP* and *SdP*, give good to perfect fits with R^2^ = 0.75–0.96 (Table 2). To visualize the goodness-of-fits of *ν_abs_(max)* and *ν_em_(max)* as functions of the Catalán solvent parameters {*SA*, *SB*, *SP*, *SdP*}, the plots of *ν_abs_(max)* and *ν_em_(max)* of **COOH-Ph-BODIPY** and **NHS-Ph-BODIPY** calculated according to Equation (5) using the estimated values of *y_0_*, *a_SA_*, *b_SB_*, *c_SP_*, and *d_SdP_* vs. the corresponding experimental *ν_abs_(max)* and *ν_em_(max)* values were drawn (Figure 2).

The Catalán model further reveals solvent properties that are mainly responsible for the observed spectral shifts. The very large (negative) estimated regression coefficients *c_SP_* compared to the *a_SA_*, *b_SB_*, and *d_SdP_* ones as well as the relatively large standard errors on the *a_SA_*, *b_SB_*, and *d_SdP_* compared to those on the *c_SP_* (Table 2) indicate that the changes of *ν_abs_(max)* and *ν_em_(max)* may primarily reflect changes in polarizability of the environment of **COOH-Ph-BODIPY** and **NHS-Ph-BODIPY** [30]. The other supporting evidence comes from the multiple linear regression analyses of the *ν_abs_(max)* and *ν_em_(max)* according to Equation (5) with {*SB*, *SP*, *SdP*}, {*SA*, *SP*, *SdP*}, and {*SA*, *SB*, *SP*} as independent variables (Table S2). The analyses give good fits with an R^2^ equal to 0.91, 0.89, and **0.72** and 0.75, 0.69, and **0.67** for *ν_abs_(max)* and *ν_em_(max)* of **COOH-Ph-BODIPY**, respectively, as well as 0.91, 0.92, and **0.78** and 0.85, 0.82, and 0.90 for *ν_abs_(max)* and *ν_em_(max)* of **NHS-Ph-BODIPY**, respectively. It is significant to note that the lowest coefficients of determination (indicated in **bold**) were obtained in case of {*SA*, *SB*, *SP*} as independent variables (except for *ν_em_(max)* of **NHS-Ph-BODIPY**), indicating that solvent dipolarity should not be neglected as an influencing factor. In contrast, the multiple linear regression analyses of the *ν_abs_(max)* and *ν_em_(max)*, according to Equation (5) with {*SA*, *SB*, *SdP*} as independent variables (Table S2), show poor fits with an R^2^ equal to 0.54 and 0.52 for *ν_abs_(max)* and *ν_em_(max)* of **COOH-Ph-BODIPY**, respectively, as well as 0.38 and 0.39 for *ν_abs_(max)* and *ν_em_(max)* of **NHS-Ph-BODIPY**, respectively. Thus, the crucial solvent property affecting the maximum absorption *ν_abs_(max)* and emission *ν_em_(max)* wavenumbers of **COOH-Ph-BODIPY** and **NHS-Ph-BODIPY** is solvent polarizability with a small contribution of solvent dipolarity.

The Lippert plots of the Stokes shifts Δ*ν* of **COOH-Ph-BODIPY** and **NHS-Ph-BODIPY** vs. orientation polarizabilities Δ*f* of the solvents chosen are presented in Figure S2. There are extremely poor linear relationships between Δ*ν* and Δ*f* with an R^2^ equal to 0.03 and 0.05, respectively. The small slopes (−68 and 56 cm^−1^, respectively) imply that the dipole moments of **COOH-Ph-BODIPY** and **NHS-Ph-BODIPY** do not change noticeably between the ground and excited states [31]. The other supporting evidence comes from the multiple linear regression analyses of the Δ*ν* within the Kamlet–Taft and Catalán models (Table S1 and Table 2), both of which show poor fits with the R^2^ equal to 0.31 and 0.29 for **COOH-Ph-BODIPY**, respectively, as well as 0.61 and 0.70 for **NHS-Ph-BODIPY**, respectively. It is interesting to note that quantum chemical calculations gave another explanation of the abovementioned trends (Section 2.3).

### 2.3. Quantum Chemical Calculations of BODIPYs

**Figure 3:**
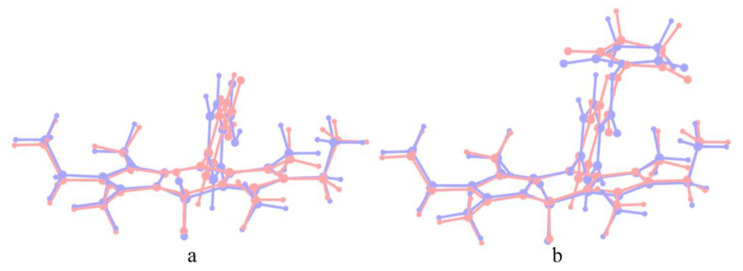
Ground S_0_ (blue) and first excited S_1_ (red) molecular geometry comparison for **COOH-Ph-BODIPY** (**a**) and **NHS-Ph-BODIPY** (**b**).

In order to gain more insight into the abovementioned relations, a series of quantum chemical calculations was performed. First, a semiempirical metadynamic screening revealed the existence of four different conformers for both **COOH-Ph-BODIPY** and **NHS-Ph-BODIPY**, of which only two were found to be non-redundant according to further DFT optimization. Those conformations differ only in the relative orientation of ethyl groups, which have limited impact on spectroscopic properties. As was later confirmed, all conformers have approximately the same excitation energies (Figure S3). It is, thus, reasonable to expect that existence of those conformers does not lead to any complications and further analysis can be performed only on the most thermodynamically stable conformer (Figure 3).

Firstly, we have obtained vibrationally resolved absorption and emission spectra of the studied molecules. Calculations were performed using the VG-FC model. As shown in the literature [32], neglect of the excited state geometry relaxation is not only a reasonable, but sometimes crucially important (for calculation convergence) assumption. The comparison of the ground and excited state geometries (Figure 3) shows that although there is a noticeable change in structure between S_0_ and S_1_ (reduction of C_7_-C_8_-C_1Ph_-C_2Ph_ dihedral angle leading to improved conjugation between BODIPY and C_8_-substituent), this change is consistent between the two studied dyes, so the general trends for relevant properties should be preserved.

**Figure 4:**
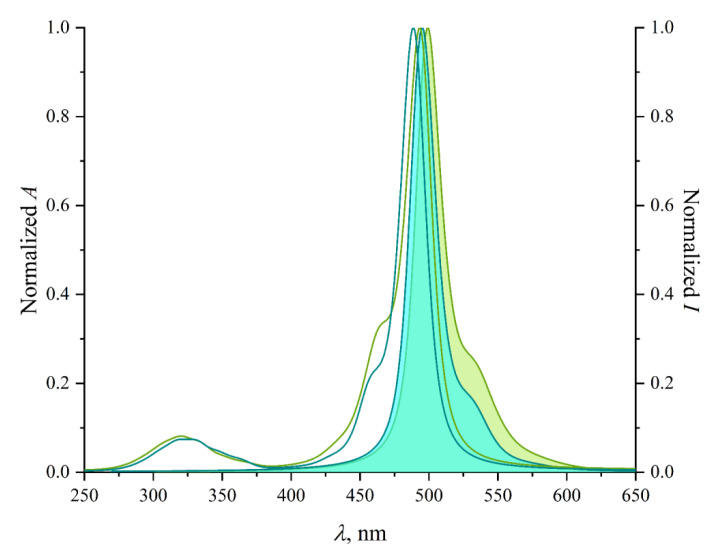
Theoretical vibrationally resolved absorption (unfilled area plot) and emission (filled area plot) spectra of **COOH-Ph-BODIPY** (blue) and **NHS-Ph-BODIPY** (green) in DMSO.

As can be seen from the VG-FC absorption and emission spectra (Figure 4), one of the preserved trends is the presence of a reasonably slight bathochromic shift resulting from the addition of succinimidyl group to BODIPY core. The presented theoretical spectra also give the same overall shape and Stokes shifts seen in experimental data (Figure 1).

Next, we have compared the results of the multiple linear regression analysis (Section 2.2) with the data obtained from the quantum chemical calculations (Table 3). In particular, relatively large (~3.6 D) ground state dipole moments of both **COOH-Ph-BODIPY** and **NHS-Ph-BODIPY** as well as large (~1.5 D) dipole moment changes upon excitation show why such Catalán solvent parameters as polarizability *SP* and dipolarity *SdP* dominate the abovementioned regressions. Moreover, the poor applicability of the Lippert relations may be explained not by a small dipole moment change upon excitation but by an indifference of the change towards solvent nature. Indeed, for both **COOH-Ph-BODIPY** and **NHS-Ph-BODIPY**, the dipole moment change is about 0.71 a.u. and for both non-polar *n*-hexane and slightly polar DMSO, the dipole moment magnitude change is about 0.68 a.u.

To gain a better understanding of the nature of this excitation, we have opted to compute the partitioned transition density matrix [33]. This technique has been used to great effect to explain the similarities and differences between compounds [34,35]. Moreover, recent papers [36] have even compared different DFT functionals in terms of the quality of their transition density matrices.

**Figure 5:**
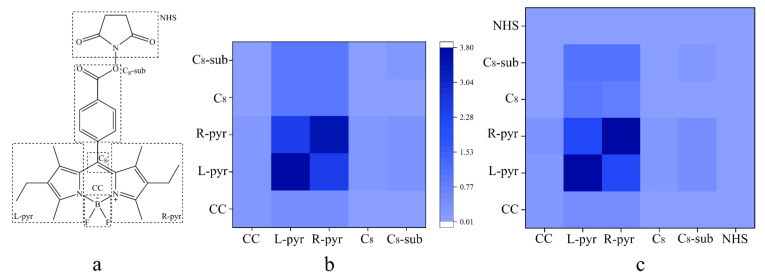
Partitioned transition density for first singlet excitation of **COOH-Ph-BODIPY** (**b**) and **NHS-Ph-BODIPY** (**c**) in DMSO and corresponding molecular segments (**a**).

We have defined five common units for **COOH-Ph-BODIPY** and **NHS-Ph-BODIPY**—two pyrrole rings (L-pyr and R-pyr), coordination center (CC), C_8_-carbon atom (C_8_), and benzoyl group (C_8_-sub)—as well as the additional one for **NHS-Ph-BODIPY**—succinimidyl group (NHS) (Figure 5a).

As can be seen from the visual representation of the matrices (Figure 5b,c), the first singlet excitation is localized mostly on the pyrrole rings and has a local character, although there is also a significant electron transfer between the rings, which, nevertheless, is symmetric. The change in the dipole moment arises due to the density transfer between the pyrrole rings as well as C_8_-carbon atom and its substituent. However, the position of the phenyl ring relative to dipyrrin is almost perpendicular due to the existence of the methyl groups in 1- and 7-positions of BODIPY core. As such, steric factors around this position should prevent any solvent molecules from getting close enough to provide a significant local impact on electron density via polarization.

Comparing two matrices, it becomes clear that the nature of the phenyl substituent does not play a strong role when it comes to the first excited state–transition density on the succinimidyl group, as well as density change between it and other units, are small. As such, as will be shown below (Section 2.4 and Section 2.5), any alterations in the absorption and emission bands positions of **COOH-Ph-BODIPY** and **NHS-Ph-BODIPY** upon conjugation with proteins will come as a result of intermolecular interactions, such as polarization due to nearby amino acids or loss of polarization induced by solvent.

### 2.4. Fluorescent Labeling of Proteins

The synthesized amine-reactive fluorescent dye **NHS-Ph-BODIPY** was used for labeling of a range of proteins, such as bovine serum albumin (BSA), PII-like protein PotN (PotN), PotA subunit of polyamine ABC transporter (PotAc), and glutamine synthetase (GS).

**Scheme 2:**
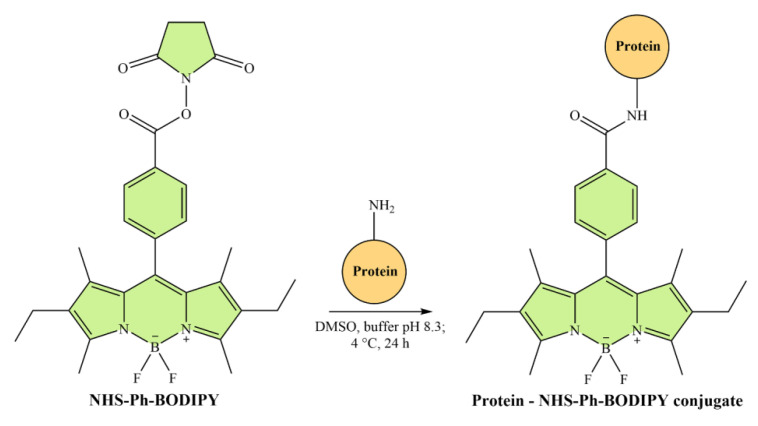
Scheme of fluorescent labeling of proteins.

The fluorescent labeling is based on a conjugation reaction between **NHS-Ph-BODIPY** possess an active succinimidyl ester moiety and a protein possess amine-containing residues (i.e., ε-amines of lysine side chains and α-amines at N-termini) to form a stable amide linkage (Scheme 2). The conjugation reaction conditions, namely slightly basic pH and low temperature, were chosen to ensure amine groups in proteins were unprotonated and to prevent hydrolysis of **NHS-Ph-BODIPY** to remove a reactive group [37]. Labeling details are given in Section 3.4.

At the first stage, the ability of **NHS-Ph-BODIPY** to label biomolecules was tested using a globular protein BSA as a model compound.

**Figure 6:**
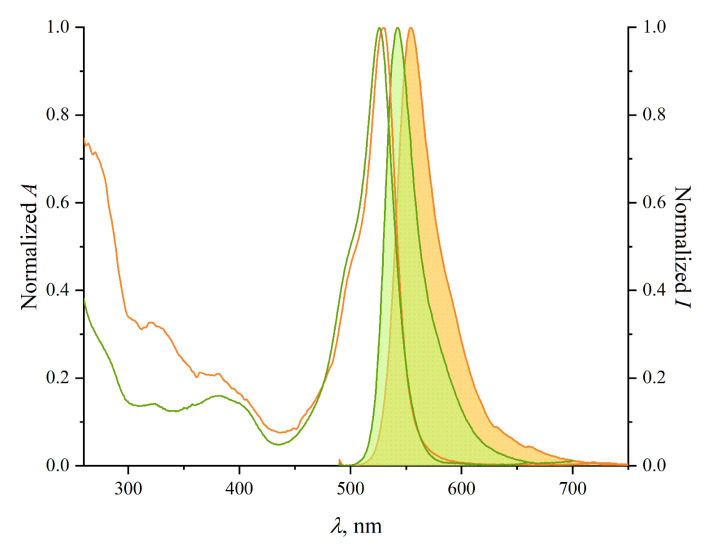
Absorption (unfilled area plot) and emission (filled area plot) spectra of **NHS-Ph-BODIPY** (green) and **BSA–NHS-Ph-BODIPY** (orange) in a DMSO–bicarbonate buffer (pH 8.3) mixture (1:9).

Absorption spectra, emission spectra, fluorescence decay curves, as well as a number of photophysical characteristics of **NHS-Ph-BODIPY** and its conjugate with BSA (**BSA–NHS-Ph-BODIPY**) were obtained in a mixture of DMSO and bicarbonate buffer with pH 8.3 (1:9) (Figure 6 and Figure S4, Table 4).

The absorption and emission spectra of **BSA–NHS-Ph-BODIPY** have several distinctive features (Figure 6). The absorption spectrum gave three bands with the maxima at 280, 380, and 530 nm, while the emission spectrum gave two bands with the maxima at 350 (not shown) and 554 nm. The new bands in the UV region are certain to relate with the absorption and emission of the protein moiety of the conjugate. The characteristic absorption and emission bands maxima of the BODIPY moiety of the conjugate are red shifted compared with the spectra of the free dye. Along with this, it is observed a decrease (about 1.5 times) of the fluorescence quantum yield of **BSA–NHS-Ph-BODIPY** in contrast to that of **NHS-Ph-BODIPY** as well as a change of the intensity decay from single exponential in case of **NHS-Ph-BODIPY** to double exponential in case of **BSA–NHS-Ph-BODIPY**. These phenomena can be explained by a dynamic quenching by aromatic amino acids located at the immediate environment of the dye in the conjugate [38,39], which is confirmed via synchronous fluorescence spectroscopy (Figure S5) and molecular docking (Section 2.5). As regards the synchronous fluorescence spectroscopy, it was found that the change from a pure BSA to a labeled BSA results in a decrease of fluorescence intensities (from 3 times in case of Δ*λ* = 15 nm to 10 times in case of Δ*λ* = 60 nm) and a blue shift (from 2 nm in case of Δ*λ* = 15 nm to 10 nm in case of Δ*λ* = 60 nm) of the bands maxima at both wavelength shifts. This suggests that the addition of **NHS-Ph-BODIPY** causes changes in the environment of tyrosine and tryptophan residues of BSA, namely an increase of its hydrophobicity [40].

Furthermore, on the basis of the absorption spectra of **NHS-Ph-BODIPY** and **BSA–NHS-Ph-BODIPY**, the degree of labeling of BSA was estimated (Section 3.7). The value amounts to 1 which means that each protein molecule possesses one fluorescent label.

At the next stage, the possibility of **NHS-Ph-BODIPY** utilization in protein analysis was examined.

**Figure 7:**
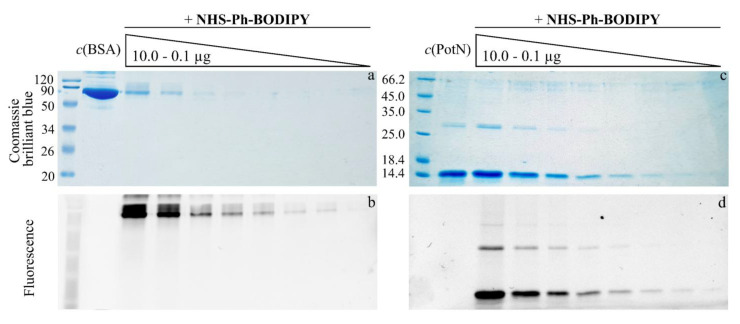
SDS-PAGE of double dilutions of BSA (MW = 66.2 kDa) (left) and PotN (MW = 13.7 kDa) (right) stained with Coomassie brilliant blue (**a**,**c**) and labeled with **NHS-Ph-BODIPY** (**b**,**d**) (each gel contains protein weight marker and unlabeled protein).

**Figure 8:**
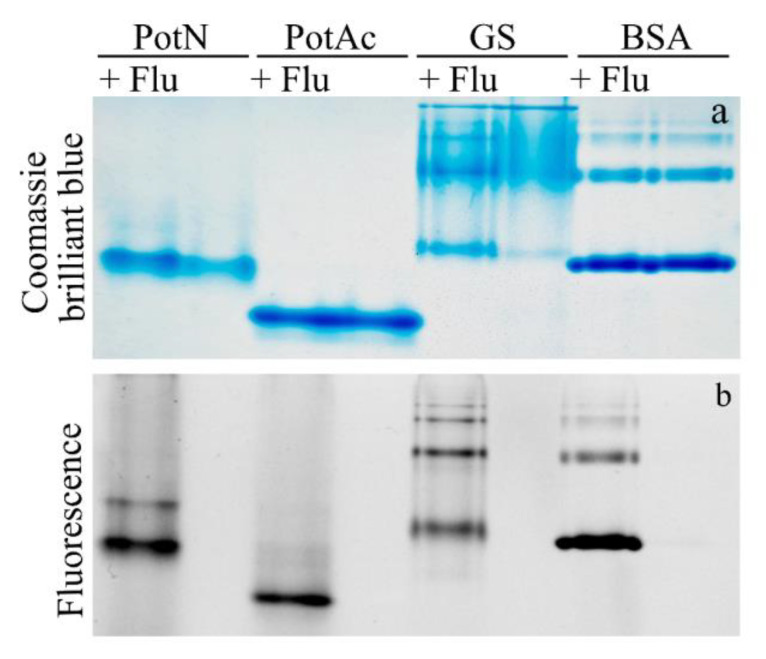
Basic native-PAGE of **NHS-Ph-BODIPY**-labeled (+ Flu) and unlabeled PotN (MW = 13.7 kDa, pI = 6.6), PotAc (MW = 18.1 kDa, pI = 5.9), GS (MW = 50.3 kDa, pI = 5.1), and BSA (MW = 66.2 kDa, pI = 5.9) visualized with Coomassie brilliant blue (**a**) and using UV transillumination (**b**) (each lane contains 10 µg of protein).

To evaluate a sensitivity of protein detection with **NHS-Ph-BODIPY**, the double dilutions (from 10.0 to 0.1 µg) of fluorescently labeled BSA and PotN were prepared and separated with SDS-PAGE. As can be seen from Figure 7, **NHS-Ph-BODIPY**-labeled BSA is detected at the concentrations up to eight times lower than Coomassie-stained BSA, while both **NHS-Ph-BODIPY**-labeled and Coomassie-stained PotNs are found at about the same concentration range. This phenomenon can be explained by a higher probability of **NHS-Ph-BODIPY** to bind lysine residues at the BSA binding site than in the PotN one (Section 2.5). Next, to evaluate whether **NHS-Ph-BODIPY** changes the charge of a protein molecule, four proteins (PotN, PotAc, GS, BSA) with various isoelectric points (pI) and molecular weights (MW) were separated under non-denaturing conditions. No difference in electrophoretic mobility of the chosen proteins was observed (Figure 8), suggesting no significant effect of **NHS-Ph-BODIPY** on the physico-chemical properties of proteins.

### 2.5. Molecular Docking of Protein–BODIPY Conjugates

To gain insight into protein–BODIPY interactions, a two-stage molecular docking of BSA and PotN with **NHS-Ph-BODIPY** was carried out.

**Figure 9:**
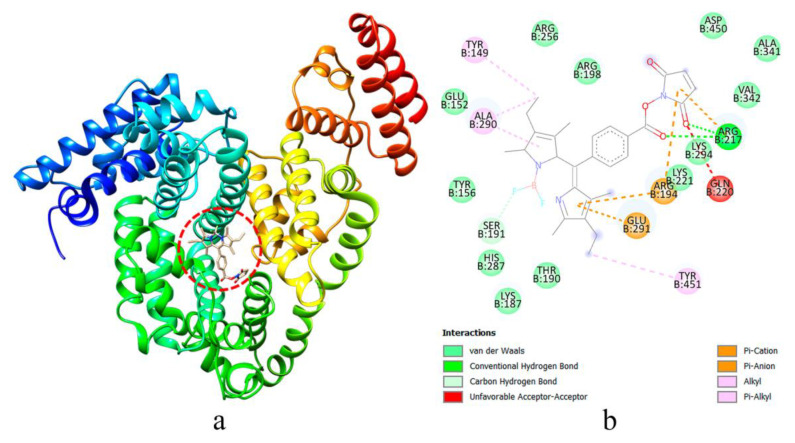
Binding site of **NHS-Ph-BODIPY** in BSA (**a**) and its amino acid composition (**b**) according to blind docking results.

At the first stage, a blind docking was performed to determine the most probable binding site of **NHS-Ph-BODIPY** in BSA. It was shown that the fluorescent dye is localized in a cavity between the IB, IIA, and IIB subdomains of the protein (Figure 9a) with a binding free energy of −26.1 kJ/mol. It is noteworthy that these subdomains are known to contain major drug binding sites [41,42,43]. The amino acid composition of the binding site of **NHS-Ph-BODIPY** in BSA is shown in Figure 9b. The binding site was found to contain three lysine residues (Lys187, Lys221, Lys294), each of which was selected for a subsequent covalent docking. In addition, it contains tyrosine (Tyr149, Tyr156, Tyr451) and tryptophan (Trp213) residues, which is consistent with the results of synchronous fluorescence spectroscopy (Section 2.4).

**Figure 10:**
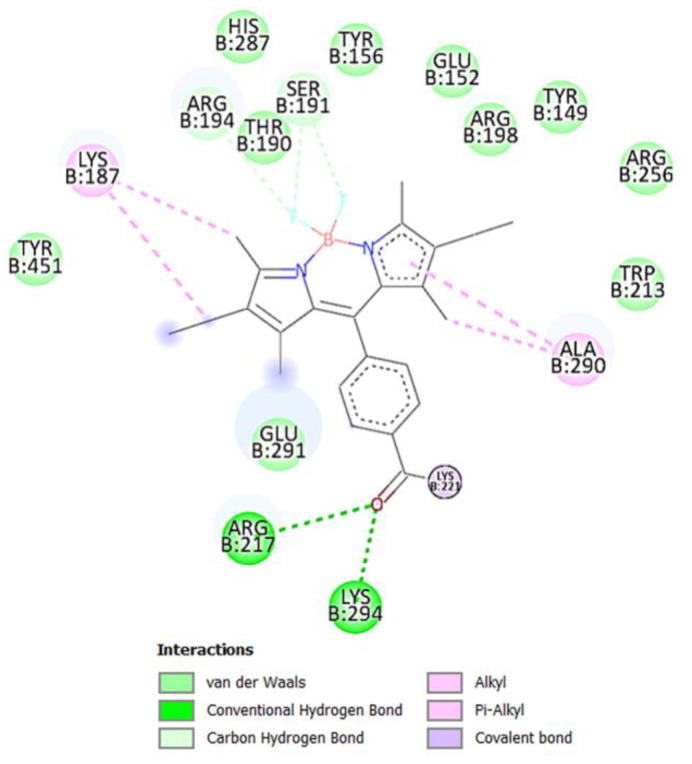
Amino acid environment of **NHS-Ph-BODIPY** in BSA according to covalent docking results of the **Lys221-BSA–NHS-Ph-BODIPY** system.

At the second stage, a covalent docking was performed to estimate a binding free energy of **NHS-Ph-BODIPY** and BSA bound together via an amide linkage through one of the lysine residues (Lys187, Lys221, Lys294) found in the binding site at the previous stage (Figure 10 and Figure S6). The binding free energies values of **Lys187-BSA–NHS-Ph-BODIPY**, **Lys221-BSA–NHS-Ph-BODIPY**, and **Lys294-BSA–NHS-Ph-BODIPY** systems being −127, −129, and −108 kJ/mol, respectively, indicate an exergonic type of the conjugation reaction. The first two systems are characterized by almost equal binding free energies, which does not allow one to get an unambiguous answer about an anchor residue involved in the conjugation. Therefore, it was estimated a distance between the tyrosine and tryptophan residues (Tyr149, Tyr156, Tyr451, Trp213) and the lysine residues bound to **NHS-Ph-BODIPY**. The distance values are 5.669 (average) and 12.201 Å for **Tyr Lys187-BSA–NHS-Ph-BODIPY** and **Trp···Lys187-BSA–NHS-Ph-BODIPY**, respectively, and 3.524 (average) and 3.357 Å for **Tyr···Lys221-BSA–NHS-Ph-BODIPY** and **Trp···Lys221-BSA–NHS-Ph-BODIPY**, respectively. Thus, a synergy of synchronous fluorescence spectroscopy and molecular docking made it possible to establish that it is Lys221 that participate in the conjugation reaction between **NHS-Ph-BODIPY** and BSA.

**Figure 11:**
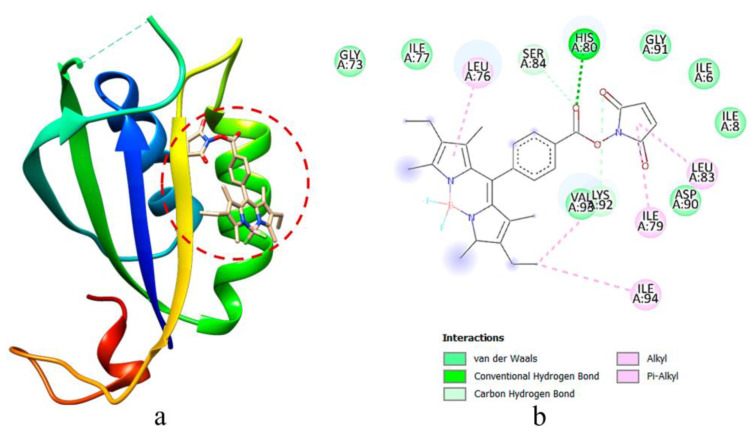
Binding site of **NHS-Ph-BODIPY** in PotN (**a**) and its amino acid composition (**b**) according to blind docking results.

A similar two-stage approach was used for molecular docking of PotN with **NHS-Ph-BODIPY**. A blind docking results shown that the fluorescent dye is localized near the B-loop of the protein (Figure 11) with a binding free energy of −31.9 kJ/mol. The binding site was found to contain one lysine residue (Lys92). A covalent docking results (Figure S7) gave a binding free energy value of −122 kJ/mol for the **Lys92-PotN–NHS-Ph-BODIPY** system.

It is noteworthy that the molecular docking results give explanation for a difference in sensitivity of BSA and PotN detection with **NHS-Ph-BODIPY** (Section 2.4). As stated above, the BSA binding site contains three lysine residues, while the PotN binding site contains only one lysine residue. Thus, lower detection limit for BSA than for PotN may be due to a higher probability of **NHS-Ph-BODIPY** to bind lysine residues at the binding site of the first protein than the second one.

## 3. Materials and Methods

### 3.1. Materials

Reagents for BODIPYs synthesis were purchased from Sigma-Aldrich (Moscow, Russia), with the exception of boron trifluoride diethyl etherate (BF_3_·OEt_2_) purchased from Merck Millipore (Burlington, MA, USA). Bovine serum albumin (BSA) was purchased from Sigma-Aldrich (Moscow, Russia), while StrepII-tagged PII-like protein PotN (PotN) and His_6_-tagged C-terminal domain of PotA subunit of polyamine ABC transporter (PotAc) from *Lentilactobacillus hilgardii* as well as StrepII-tagged glutamine synthetase (GS) from *Bacillus subtilis* were obtained as described earlier [44,45]. Organic solvents were bought from Sigma-Aldrich (Moscow, Russia), EKOS-1, and Khimmed. Substances for buffers preparation were bought from Sigma-Aldrich and Reakhim (Moscow, Russia).

Dialysis tubing MD25 with 8.0–14.0 kDa molecular weight cut-off was obtained from a commercial source and prepared for use according to the procedure [46].

### 3.2. Instruments

^1^H and ^11^B nuclear magnetic resonance (NMR) spectra were recorded on a Bruker Avance III 500 NMR spectrometer (Billerica, MA, USA) with operating frequencies of 500.17 for ^1^H and 160.48 MHz for ^11^B. Deuterated chloroform was chosen as a solvent for samples preparation. Tetramethylsilane was used as an internal reference (*δ* = 0.00 ppm) for ^1^H NMR studies, while BF_3_·OEt_2_ was used as an external reference (*δ* = 0.00 ppm) for ^11^B NMR studies. The following abbreviations are used to designate peak multiplicities and descriptors: s for singlet, d for doublet, t for triplet, and q for quadruplet. Infrared (IR) spectra were obtained by means of a Shimadzu IRAffinity-1 Fourier transform IR spectrophotometer (Billerica, MA, USA) equipped with a Specac Quest ATR Diamond GS10800-B accessory in the mid-infrared (400–4000 cm^−1^) region. The following abbreviations are used to designate signal intensities: w for weak, m for medium, s for strong, br for broad, and sh for sharp. Matrix-assisted laser desorption/ionization (MALDI) time of flight (TOF) mass spectra (MS) were recorded on a Shimadzu AXIMA Confidence MALDI TOF-TOF mass spectrometer in positive ion reflectron mode.

Ultraviolet-visible (UV-Vis) spectroscopy experiments were performed on an Aquilon SF-104 spectrophotometer (Waltham, MA, USA). Absorption spectra were measured in the range of 190–800 nm. Fluorescence spectroscopy experiments were performed on an Agilent Cary Eclipse fluorescence spectrophotometer (Santa Clara, CA, USA). Steady-state emission spectra were measured in the ranges of 270–400 and 490–800 nm with excitation wavelengths of 260 and 480 nm, respectively. Synchronous emission spectra were measured in the range of 250–350 nm with wavelength shifts Δ*λ* of 15 and 60 nm. Excitation and emission slit widths values were 5.0 nm. Time-resolved fluorescence spectroscopy experiments were carried out by means of a PicoQuant FluoTime 300 high performance fluorescence lifetime and steady state spectrometer with a PicoQuant PLS 450 sub-nanosecond pulsed light-emitting diode as an excitation source. An instrument response function of a system was measured with a stray light signal of a dilute colloidal silica suspension (LUDOX^®^). Fluorescence decay curves were measured at the maximum of the emission peaks and fluorescence lifetimes were obtained by reconvolution of the decay curves using a PicoQuant EasyTau 2 software package. Standard quartz cuvettes with 10 mm light path were used for all experiments.

Labeled proteins were separated with sodium dodecyl sulfate–polyacrylamide gel electrophoresis (SDS-PAGE) and basic native–polyacrylamide gel electrophoresis (basic native-PAGE) [47], visualized with Bio-Rad ChemiDoc XRS+ System in trans-UV mode and then stained with Coomassie brilliant blue.

### 3.3. Synthesis of Reactive BODIPY

4-(2,6-Diethyl-4,4-difluoro-1,3,5,7-tetramethyl-4-bora-3a,4a-diaza-*s*-indacene-8-yl)-benzoic acid (**COOH-Ph-BODIPY**). Synthesis was carried out using a mechanochemical approach [48] (Scheme 1, Figures S8–S11). 3-Ethyl-2,4-dimethylpyrrole (0.3 mL, 2.2 mmol, 2 equiv.), 4-carboxybenzaldehyde (167.0 mg, 1.1 mmol, 1 equiv.), trifluoroacetic acid (TFA) (1 drop), p-chloranil (407.0 mg, 1.7 mmol, 1.5 equiv.), and a small amount of dichloromethane (DCM) as a binder additive were mixed with a pestle and mortar. The mixture was ground for 10 min until a thick dark purple paste with green metallic luster was formed. After that, triethylamine (Et_3_N) (1.2 mL, 9.0 mmol, 8 equiv.) and dropwise BF_3_·OEt_2_ (1.3 mL, 10.3 mmol, 9 equiv.) were added. The mixture was ground for 10 min until a thick dark purple paste with green metallic luster was formed. After that, the resulting paste was dissolved in DCM–ethyl acetate (1:1) mixture (150 mL), filtered through a Buchner funnel, and washed with brine (3 × 50 mL). The organic solvents were removed in vacuo, and the crude residue was purified by column chromatography on silica gel using diethyl ester–hexane (1:1) mixture. After the removal of eluent in vacuo, 131.8 mg of dark red powder of the desired product was obtained, resulting in 28% yield. ^1^H NMR: *δ* (ppm) 8.23 (d, 2H, *J* = 7.9 Hz, –C_aryl_–H), 7.44 (d, 2H, *J* = 7.8 Hz, –C_aryl_–H), 2.53 (s, 6H, –CH_3_), 2.30 (q, 4H, *J* = 7.6 Hz, –CH_2_–), 1.26 (s, 6H, –CH_3_), 0.98 (t, 6H, *J* = 7.6 Hz, –CH_3_). ^11^B NMR: *δ* (ppm) 0.78 (t, 1B, *J* = 33.9 Hz, –BF_2_–). IR: *ν* (cm^−1^) 2963–2870 (m, br, C_aliphatic_–H, O–H), 1684 (s, sh, C=O), 1473 (m, N–B), 1261 (s, sh, C–O), 1112 (m, B–F), 1073 (m, B–F). MS (MALDI TOF): *m*/*z* calculated for C_24_H_27_BF_2_N_2_O_2_^+^ [M]^+^ 424.21, found to be 424.99.

Succinimidyl ester of 4-(2,6-diethyl-4,4-difluoro-1,3,5,7-tetramethyl-4-bora-3a,4a-diaza-*s*-indacene-8-yl)-benzoic acid (**NHS-Ph-BODIPY**). Synthesis was carried out on the basis of the previously described procedure [25] (Scheme 1, Figures S8–S11). After the synthesis and all workups, 15.3 mg of orange-red powder of the desired product was obtained resulting in 41% yield. ^1^H NMR: *δ* (ppm) 8.26 (d, 2H, *J* = 7.9 Hz, –C_aryl_–H), 7.49 (d, 2H, *J* = 7.9 Hz, –C_aryl_–H), 2.96 (d, 4H, *J* = 7.8 Hz, –CH_2_–_succinimide_), 2.53 (s, 6H, –CH_3_), 2.30 (q, 4H, *J* = 7.6 Hz, –CH_2_–), 1.27 (s, 6H, –CH_3_), 0.98 (t, 6H, *J* = 7.5 Hz, –CH_3_). ^11^B NMR: *δ* (ppm) 0.76 (t, 1B, *J* = 34.0 Hz, –BF_2_–). IR: *ν* (cm^−1^) 2964–2853 (m, br, C_aliphatic_–H), 1800 (w, C=O_succinimide_), 1767 (m, C=O_succinimide_), 1739 (s, C=O), 1473 (m, N–B), 1117 (m, B–F), 1064 (m, B–F). MS (MALDI TOF): *m*/*z* calculated for C_28_H_30_BF_2_N_3_O_4_^+^ [M]^+^ 521.23, found to be 522.06.

### 3.4. Fluorescent Labeling of Proteins

Fluorescent labeling of proteins was carried out on the basis of the standard amine-reactive probe labeling protocol [49] (Scheme 2). A solution of **NHS-Ph-BODIPY** (8 equiv.) in dimethyl sulfoxide (DMSO) was slowly added to a solution of protein (1 equiv.) in bicarbonate or phosphate buffer with pH 8.3. After a thorough stirring, the mixture was incubated at 4 °C for 24 h. After completion of the reaction, the mixture was dialyzed against 70% ethanol (three times) and then distilled water (three times) to give a pure labeled protein solution.

### 3.5. Determination of Photophysical Characteristics

The fluorescence quantum yields Φ of the compounds investigated were estimated by comparison with Rhodamine 6G as a standard of known fluorescence quantum yield (Φ = 0.91 in ethanol [50]) using Equation (2) [50]:

(2) $$\Phi_{x}=\Phi_{st}\frac{S_{x}}{S_{st}}\frac{1-10^{-A_{st}}}{1-10^{-A_{x}}}\frac{n_{x}^{2}}{n_{st}^{2}}$$

where *S* is the integrated area under the emission spectrum, *A* is the absorbance at the excitation wavelength, and *n* is the refractive index of the solvent. The subscripts *x* and *st* refer to the unknown and reference solutions, respectively.

The radiative *k_r_* and non-radiative *k_nr_* rate constants were calculated using Equations (3) and (4) [26], respectively:

(3) $$k_{r}=\frac{\Phi }{\tau }\;$$

(4) $$k_{nr}=\frac{1}{\tau }-k_{r}$$

where *τ* is the fluorescence lifetime.

### 3.6. Description of Solvent Effects via Multiparameter Approach

In order to describe solvent effects on spectral characteristics of the compounds investigated, a multiparameter approach using the Catalán equation (Equation (5)) [28] was applied:

(5) $$y=y_{0}+a_{SA}SA+b_{SB}SB+c_{SP}SP+d_{SdP}SdP$$

where *y* is the value of a solvent-dependent physicochemical property in a given solvent, *y_0_* is the statistical quantity corresponding to the value of a solvent-dependent physicochemical property in the gas phase or in an inert solvent, *SA* is the empirical parameter of solvent hydrogen-bond donor acidity, *SB* is the empirical parameter of solvent hydrogen-bond acceptor basicity, *SP* is the empirical parameter of solvent polarizability, *SdP* is the empirical parameter of solvent dipolarity, and *a* − *d* are the regression coefficients describing the sensitivity of a physicochemical property *y* to the different solute/solvent interaction mechanisms.

### 3.7. Determination of Degree of Labeling

The degrees of labeling of the proteins investigated were estimated using Equation (6) [49]:

(6) $$DOL=\frac{A_{\max conj}}{\varepsilon_{\max dye}}\frac{\varepsilon_{\max prot}}{A_{280\;conj}-A_{\max conj}\frac{A_{280\;dye}}{A_{max\;dye}}}$$

where *DOL* is the degree of labeling, *A_max conj_* and *A_max dye_* are the absorbances of the conjugated dye and the free dye at the maximum absorption wavelength, respectively, *ε_max prot_* and *ε_max dye_* are the molar absorption coefficients of the free protein and the free dye at the absorption wavelength, respectively, and *A_280 conj_* and *A_280 dye_* are the absorbances of the labeled protein and maximum the free dye at 280 nm, respectively.

### 3.8. Quantum Chemical Calculations Procedure

The conformational search for **COOH-Ph-BODIPY** and **NHS-Ph-BODIPY** was performed in a metadynamic approximation. The calculations were carried out by means of the xtb program [51] with the CREST add-on [52]. The more accurate GFN2-xTB method was chosen along with the iMTD-GC algorithm. The conformational screening revealed four conformers for **COOH-Ph-BODIPY** and four conformers for **NHS-Ph-BODIPY**. The lowest energy conformers were chosen for further DFT and TDDFT study. Each conformer was optimized at the CAM-B3LYP/6-31G(d,p) level, which provides a balanced description of both ground and excited states [53]. For each resulting structure, ten excitation vertical absorption spectra were computed. For the first three excitations, gradients as well as vertical gradient Franck-Condon (VG-FC) vibronic spectra within the TD formalism as implemented in the FCclasses 3.0 program [54] were also obtained. Vibronic spectra calculations used ground state vibrational frequencies, vertical excitation energies computed at ground state geometries and excited state gradients computed at the same geometries. All empirical parameters pertaining to the VG-FC algorithm, such as number of points for the fast Fourier transform (FFT), were directly estimated by the FCclasses program. All vibronic spectra are broadened with Gaussian functions with the constant width of 322 cm^−1^. This value was chosen because it gives the best agreement between experimental and theoretical spectra of fully unsubstituted BODIPY. All calculations, unless otherwise mentioned, were performed within the CPCM model with DMSO parameters using the ORCA 5.0 program suite [55,56,57]. Quantities of interest, such as ground and excited state dipole moments as well as transition density matrix elements, were computed using the latest version of the Multiwfn program [58]. A visualization of the quantum chemical calculations results were made by means of both the Chemcraft 1.8 [59] and the VMD [60] programs.

### 3.9. Molecular Docking Procedure

The molecular docking of the proteins–BODIPY conjugates was performed in two stages. At the first stage, a blind docking was carried out by means of the Autodock 4.2 program [61]. The crystal structures of the proteins under study were taken from the Protein Data Bank: BSA [62] and PotN [63]. The structure of **NHS-Ph-BODIPY** was obtained by virtue of geometry optimization (Section 3.8). The calculations were performed for a 126 Å × 126 Å × 126 Å grid with BSA in its center with a step of 0.7 Å and a 90 Å × 126 Å × 126 Å grid with PotN in its center with a step of 0.4 Å. Each docking experiment included 50 runs with a maximum of 25 million energy evaluations using the Lamarckian genetic algorithm [64]. Conformations of the protein–BODIPY conjugates with minimum energies were assumed as the most stable ones. At the second stage, a covalent docking was carried out by means of the CovDock program [65]. On the basis of the blind docking results, a covalent binding of **NHS-Ph-BODIPY** and lysine residues (imine condensation pre-defined reaction) at the most energetically favorable binding site of the proteins was calculated. Conformations of the protein–BODIPY conjugates with minimum energies were again assumed as the most stable ones. A visualization of the molecular docking results were made by means of the UCSF Chimera program [66].

## 4. Conclusions

The development of fluorescent dyes reactive towards principal functional groups of protein is a promising area of modern chemistry. Therefore, we carried out a thorough investigation of spectroscopic and photophysical properties as well as solvatochromic behavior of the synthesized amine-reactive fluorescent dye **NHS-Ph-BODIPY** and its non-reactive precursor **COOH-Ph-BODIPY** by means of ultraviolet-visible and fluorescence spectroscopy supported by quantum chemical calculations that allowed us to better understand some specific aspects of their functioning in solutions. An inherent stable bright green fluorescence of **NHS-Ph-BODIPY** excited us to use this dye for fluorescent labeling of a number of proteins of various nature. **NHS-Ph-BODIPY** was demonstrated to act at least at the same level or even several times better than Coomassie brilliant blue when detecting proteins via SDS-PAGE. Thus, the sum of the experimental and theoretical results suggests that the synthesized amine-reactive fluorescent dye **NHS-Ph-BODIPY** is a promising fluorescent label for fluorescent immunoassay, cellular imaging and in vivo imaging, flow cytometry, immunohistochemical staining, etc.

## Figures and Tables

**Table 1:** Photophysical characteristics of **COOH-Ph-BODIPY** and **NHS-Ph-BODIPY** in organic solvents.

	Solvent	*λ_abs max_*, nm	*λ_em max_*, nm	Δ*ν*, cm^−1^	*ε*, M^−1^·cm^−1^	*Φ*	*τ*, ns	*k_r_*, ns^−1^	*k_nr_*, ns^−1^
**COOH-Ph-BODIPY**									
1	*n*-Hexane	527	543	559	98,500	0.61	3.23	0.19	0.12
2	Toluene	529	549	689	99,500	0.61	4.09	0.15	0.09
3	Ethyl acetate	524	541	600	118,500	0.73	4.27	0.17	0.06
4	Chloroform	529	546	589	91,400	0.56	4.32	0.13	0.10
5	DCM	528	547	658	61,500	0.38	3.76	0.10	0.16
6	Acetone	523	542	670	51,900	0.32	3.66*	0.09	0.19
7	DMSO	525	541	563	70,900	0.44	4.57	0.09	0.12
8	Acetonitrile	521	538	606	59,200	0.36	4.25*	0.09	0.15
9	*n*-Propanol	523	539	568	70,400	0.43	4.76*	0.09	0.12
10	Ethanol	523	539	568	56,700	0.35	4.85*	0.07	0.13
**NHS-Ph-BODIPY**									
1	*n*-Hexane	529	547	622	69,800	0.35	3.17	0.11	0.21
2	Toluene	532	552	681	61,900	0.43	4.02	0.11	0.14
3	Ethyl acetate	527	544	593	56,900	0.56	3.70	0.15	0.12
4	Chloroform	531	550	651	73,100	0.46	3.64	0.13	0.15
5	DCM	530	550	686	55,000	0.40	3.75	0.11	0.16
6	Acetone	526	545	663	55,200	0.35	3.23	0.11	0.20
7	DMSO	529	548	655	59,600	0.47	4.14	0.11	0.13
8	Acetonitrile	525	544	665	46,500	0.39	3.43	0.11	0.18
9	*n*-Propanol	527	546	660	52,600	0.49	3.60	0.13	0.14
10	Ethanol	527	545	627	54,000	0.38	3.85	0.10	0.16

**Table 2:** Regression coefficients *y_0_*, *a* − *d* and coefficients of determination R^2^ for multiple linear regression analysis of maximum absorption *ν_abs_(max)* and emission *ν_em_(max)* wavenumbers and Stokes shifts Δ*ν* of **COOH-Ph-BODIPY** and **NHS-Ph-BODIPY** as a function of Catalán solvent scale parameters {*SA*, *SB*, *SP*, *SdP*}.

	*y*_0_	*a_SA_*	*b_SB_*	*c_SP_*	*d_SdP_*	R^2^
**COOH-Ph-BODIPY**						
*ν_abs_(max)*, cm^−1^	19,553 ± 129	−108 ± 113	131 ± 77	−988 ± 190	203 ± 54	0.93
*ν_em_(max)*, cm^−1^	18,901 ± 291	13 ± 254	184 ± 172	−924 ± 482	157 ± 122	0.75
Δ*ν*, cm^−1^	653 ± 189	−122 ± 165	−53 ± 112	−64 ± 278	46 ± 79	0.29
**NHS-Ph-BODIPY**						
*ν_abs_(max)*, cm^−1^	19,461 ± 76	−167 ± 66	100 ± 45	−940 ± 112	150 ± 32	0.96
*ν_em_(max)*, cm^−1^	18,912 ± 104	−258 ± 91	204 ± 62	−1064 ± 154	81 ± 44	0.94
Δ*ν*, cm^−1^	549 ± 73	91 ± 63	−104 ± 43	124 ± 107	68 ± 31	0.70

**Table 3:** Ground S_0_ and first excited S_1_ total dipole moments ||*µ_GS_*|| and ||*µ_TD_*|| of **COOH-Ph-BODIPY** and **NHS-Ph-BODIPY**, difference between their absolute values, and absolute value of their difference in *n*-hexane and DMSO.

Compound	Solvent	||*µ_GS_*||, a.u.	||*µ_TD_*||, a.u.	||*µ_GS_*|| − ||*µ_TD_*||, a.u.	||*µ_GS_* − *µ_TD_*||, a.u.
**COOH-Ph-BODIPY**	*n*-Hexane	1.405	0.724	0.681	0.714
	DMSO	1.658	0.975	0.683	0.711
**NHS-Ph-BODIPY**	*n*-Hexane	1.990	1.310	0.679	0.718
	DMSO	2.336	1.659	0.678	0.711

**Table 4:** Photophysical characteristics of **NHS-Ph-BODIPY** and **BSA–NHS-Ph-BODIPY** in a DMSO–bicarbonate buffer (pH 8.3) mixture (1:9).

	*λ_abs max_*, nm	*λ_em max_*, nm	Δ*ν*, cm^−1^	*Φ*	*τ*_1_, ns	*τ*_2_, ns
**NHS-Ph-BODIPY**	526	542	561	0.41	4.96	–
**BSA–NHS-Ph-BODIPY**	280	350	7143	0.30	2.79	8.00
	530	554	817			

## Supplementary Materials

The following are available online at https://www.mdpi.com/article/10.3390/molecules27227911/s1, Figure S1: Fluorescence decay curves of **COOH-Ph-BODIPY** and **NHS-Ph-BODIPY** in DMSO and *n*-propanol; Figure S2: Lippert plots for **COOH-Ph-BODIPY** and **NHS-Ph-BODIPY**; Figure S3: First 10 singlet excitation energies and their oscillator strengths for each of two conformers of **COOH-Ph-BODIPY** and **NHS-Ph-BODIPY** according to TD-DFT calculations; Figure S4: Fluorescence decay curves of **NHS-Ph-BODIPY** and **BSA–NHS-Ph-BODIPY** in DMSO–bicarbonate buffer (pH 8.3) mixture (1:9); Figure S5: Synchronous emission spectra of BSA and **BSA–NHS-Ph-BODIPY** in DMSO–bicarbonate buffer (pH 8.3) mixture (1:9); Figures S6 and S7: Amino acid environment of **NHS-Ph-BODIPY** in BSA and PotN according to covalent docking results of **Lys187-BSA–NHS-Ph-BODIPY**, **Lys221-BSA–NHS-Ph-BODIPY**, **Lys294-BSA–NHS-Ph-BODIPY**, and **Lys92-PotN–NHS-Ph-BODIPY** systems; Figures S8–S11: ^1^H NMR, ^11^B NMR, IR, and MS spectra of **COOH-Ph-BODIPY** and **NHS-Ph-BODIPY**; Tables S1 and S2: Regression coefficients *y_0_*, *a*–*d*, and coefficients of determination R^2^ for multiple linear regression analysis of maximum absorption *ν_abs_(max)* and emission *ν_em_(max)* wavenumbers and Stokes shifts Δ*ν* of **COOH-Ph-BODIPY** and **NHS-Ph-BODIPY** as a function of Kamlet–Taft {*α*, *β*, *π**} and Catalán {*SB*, *SP*, *SdP*}, {*SA*, *SP*, *SdP*}, {*SA*, *SB*, *SdP*}, and {*SA*, *SB*, *SP*} solvent scale parameters; S1: Description of solvent effects.

## References

[1] Lavis L.D., Raines R.T. (2008). Bright Ideas for Chemical Biology. ACS Chem. Biol..

[2] Specht E.A., Braselmann E., Palmer A.E. (2017). A Critical and Comparative Review of Fluorescent Tools for Live-Cell Imaging. Annu. Rev. Physiol..

[3] Kaur P., Singh K. (2019). Recent Advances in the Application of BODIPY in Bioimaging and Chemosensing. J. Mater. Chem. C.

[4] Nakamura M., Tsumoto K., Ishimura K., Kumagai I. (2002). Detection of Biotinylated Proteins in Polyacrylamide Gels Using an Avidin-Fluorescein Conjugate. Anal. Biochem..

[5] Hama Y., Urano Y., Koyama Y., Kamiya M., Bernardo M., Paik R.S., Shin I.S., Paik C.H., Choyke P.L., Kobayashi H. (2007). A Target Cell-Specific Activatable Fluorescence Probe for In Vivo Molecular Imaging of Cancer Based on a Self-Quenched Avidin-Rhodamine Conjugate. Cancer Res..

[6] Beija M., Afonso C.A.M., Martinho J.M.G. (2009). Synthesis and Applications of Rhodamine Derivatives as Fluorescent Probes. Chem. Soc. Rev..

[7] Ferreira S.Z., Carneiro H.C., Lara H.A., Alves R.B., Resende J.M., Oliveira H.M., Silva L.M., Santos D.A., Freitas R.P. (2015). Synthesis of a New Peptide-Coumarin Conjugate: A Potential Agent against Cryptococcosis. ACS Med. Chem. Lett..

[8] Ni Y., Wu J. (2014). Far-Red and Near Infrared BODIPY Dyes: Synthesis and Applications for Fluorescent pH Probes and Bio-Imaging. Org. Biomol. Chem..

[9] Kowada T., Maeda H., Kikuchi K. (2015). BODIPY-Based Probes for the Fluorescence Imaging of Biomolecules in Living Cells. Chem. Soc. Rev..

[10] Antina E., Bumagina N., Marfin Y., Guseva G., Nikitina L., Sbytov D., Telegin F. (2022). BODIPY Conjugates as Functional Compounds for Medical Diagnostics and Treatment. Molecules.

[11] Marek M., Kaiser K., Gruber H.J. (1997). Biotin-Pyrene Conjugates with Poly(Ethylene Glycol) Spacers are Convenient Fluorescent Probes for Avidin and Streptavidin. Bioconjug. Chem..

[12] Carreon J.R., Stewart K.M., Mahon K.P., Shin S., Kelley S.O. (2007). Cyanine Dye Conjugates as Probes for Live Cell Imaging. Bioorg. Med. Chem. Lett..

[13] Loudet A., Burgess K. (2007). BODIPY Dyes and Their Derivatives: Syntheses and Spectroscopic Properties. Chem. Rev..

[14] Boens N., Leen V., Dehaen W. (2012). Fluorescent Indicators Based on BODIPY. Chem. Soc. Rev..

[15] Rezende L.C.D., Emery F.S. (2013). A Review of the Synthetic Strategies for the Development of BODIPY Dyes for Conjugation with Proteins. Orbital Electron. J. Chem..

[16] Dilek O., Bane S.L. (2009). Synthesis, Spectroscopic Properties and Protein Labeling of Water Soluble 3,5-Disubstituted Boron Dipyrromethenes. Bioorg. Med. Chem. Lett..

[17] Wang D., Fan J., Gao X., Wang B., Sun S., Peng X. (2009). Carboxyl BODIPY Dyes from Bicarboxylic Anhydrides: One-Pot Preparation, Spectral Properties, Photostability, and Biolabeling. J. Org. Chem..

[18] Poirel A., Retailleau P., de Nicola A., Ziessel R. (2014). Synthesis of Water-Soluble Red-Emitting Thienyl-BODIPYs and Bovine Serum Albumin Labeling. Chem. Eur. J..

[19] Kim D., Ma D., Kim M., Jung Y., Kim N.H., Lee C., Cho S.W., Park S., Huh Y., Jung J. (2017). Fluorescent Labeling of Protein Using Blue-Emitting 8-Amino-BODIPY Derivatives. J. Fluoresc..

[20] Ksenofontova K.V., Ksenofontov A.A., Khodov I.A., Rumyantsev E.V. (2019). Novel BODIPY-Conjugated Amino Acids: Synthesis and Spectral Properties. J. Mol. Liq..

[21] Amorim V.G., Melo S.M.G., Leite R.F., Coutinho P.A., da Silva S.M.P., Silva A.R., Amorim F.G., Pires R.G.W., Coitinho J.B., Emery F.S. (2020). Synthesis and Characterization of Two Novel Red-Shifted Isothiocyanate BODIPYs and Their Application in Protein Conjugation. Dyes Pigment..

[22] Jeon S., Kim T.-I., Jin H., Lee U., Bae J., Bouffard J., Kim Y. (2020). Amine-Reactive Activated Esters of meso-CarboxyBODIPY: Fluorogenic Assays and Labeling of Amines, Amino Acids, and Proteins. J. Am. Chem. Soc..

[23] Ksenofontova K.V., Ksenofontov A.A., Khodov I.A., Rumyantsev E.V. (2020). Synthesis and Study of Spectral Properties of Amino Acids-BODIPY Conjugates. Izv. Vyssh. Uchebn. Zaved. Khim. Khim. Tekhnol..

[24] Raskolupova V.I., Popova T.V., Zakharova O.D., Nikotina A.E., Abramova T.V., Silnikov V.N. (2021). Human Serum Albumin Labelling with a New BODIPY Dye Having a Large Stokes Shift. Molecules.

[25] Brellier M., Duportail G., Baati R. (2010). Convenient Synthesis of Water-Soluble Nitrilotriacetic Acid (NTA) BODIPY Dyes. Tetrahedron Lett..

[26] Lakowicz J.R. (2006). Principles of Fluorescence Spectroscopy.

[27] Kamlet M.J., Abboud J.L.M., Abraham M.H., Taft R.W. (1983). Linear Solvation Energy Relationships. 23. A Comprehensive Collection of the Solvatochromic Parameters, π*, α, and β, and Some Methods for Simplifying the Generalized Solvatochromic Equation. J. Org. Chem..

[28] Catalán J. (2009). Toward a Generalized Treatment of the Solvent Effect Based on Four Empirical Scales: Dipolarity (SdP, a New Scale), Polarizability (SP), Acidity (SA), and Basicity (SB) of the Medium. J. Phys. Chem. B.

[29] Mataga N., Kaifu Y., Koizumi M. (1956). Solvent Effects upon Fluorescence Spectra and the Dipolemoments of Excited Molecules. Bull. Chem. Soc. Jpn..

[30] Boens N., Leen V., Dehaen W., Wang L., Robeyns K., Qin W., Tang X., Beljonne D., Tonnelé C., Paredes J.M. (2012). Visible Absorption and Fluorescence Spectroscopy of Conformationally Constrained, Annulated BODIPY Dyes. J. Phys. Chem. A.

[31] Cieślik-Boczula K., Burgess K., Li L., Nguyen B., Pandey L., de Borggraeve W.M., van der Auweraer M., Boens N. (2009). Photophysics and Stability of Cyano-Substituted Boradiazaindacene Dyes. Photochem. Photobiol. Sci..

[32] Banerjee S., Stüker T., Saalfrank P. (2015). Vibrationally Resolved Optical Spectra of Modified Diamondoids Obtained from Time-Dependent Correlation Function Methods. Phys. Chem. Chem. Phys..

[33] McWeeny R. (1960). Some Recent Advances in Density Matrix Theory. Rev. Mod. Phys..

[34] Tariq R., Khera R.A., Rafique H., Azeem U., Naveed A., Ayub A.R., Iqbal J. (2021). Computational and Theoretical Study of Subphthalocyanine Based Derivatives by Varying Acceptors to Increase the Efficiency of Organic Solar Cells. Comput. Theor. Chem..

[35] Tretiak S., Igumenshchev K., Chernyak V. (2005). Exciton Sizes of Conducting Polymers Predicted by Time-Dependent Density Functional Theory. Chem. Rev..

[36] Titov E. (2021). On the Low-Lying Electronically Excited States of Azobenzene Dimers: Transition Density Matrix Analysis. Molecules.

[37] Hermanson G.T. (2013). Bioconjugate Techniques.

[38] Jones Brunette A.M., Farrens D.L. (2014). Distance Mapping in Proteins Using Fluorescence Spectroscopy: Tyrosine, Like Tryptophan, Quenches Bimane Fluorescence in a Distance-Dependent Manner. Biochemistry.

[39] Karolin J., Johansson L.B.-A., Strandberg L., Ny T. (1994). Fluorescence and Absorption Spectroscopic Properties of Dipyrrometheneboron Difluoride (BODIPY) Derivatives in Liquids, Lipid Membranes, and Proteins. J. Am. Chem. Soc..

[40] Wang Y.-Q., Zhang H.-M., Zhang G.-C. (2006). Studies of the Interaction between Palmatine Hydrochloride and Human Serum Albumin by Fluorescence Quenching Method. J. Pharm. Biomed. Anal..

[41] Ramezani F., Rafii-Tabar H. (2015). An In-Depth View of Human Serum Albumin Corona on Gold Nanoparticles. Mol. Biosyst..

[42] Zsila F. (2013). Subdomain IB is the Third Major Drug Binding Region of Human Serum Albumin: Toward the Three-Sites Model. Mol. Pharm..

[43] Fanali G., Di Masi A., Trezza V., Marino M., Fasano M., Ascenzi P. (2012). Human Serum Albumin: From Bench to Bedside. Mol. Asp. Med..

[44] Iskhakova Z.I., Zhuravleva D.E., Heim C., Hartmann M.D., Laykov A.V., Forchhammer K., Kayumov A.R. (2022). PotN Represents a Novel Energy-State Sensing PII Subfamily, Occurring in Firmicutes. FEBS J..

[45] Heinrich A., Woyda K., Brauburger K., Meiss G., Detsch C., Stülke J., Forchhammer K. (2006). Interaction of the Membrane-Bound GlnK-AmtB Complex with the Master Regulator of Nitrogen Metabolism TnrA in Bacillus subtilis. J. Biol. Chem..

[46] McPhie P., Jakoby W.B. (1971). [4] Dialysis. Enzyme Purification and Related Techniques.

[47] Laemmli U.K. (1970). Cleavage of Structural Proteins during the Assembly of the Head of Bacteriophage T4. Nature.

[48] Jameson L.P., Dzyuba S.V. (2013). Expeditious, Mechanochemical Synthesis of BODIPY Dyes. Beilstein J. Org. Chem..

[49] Haugland R.P., Davis W.C. (1995). Coupling of Monoclonal Antibodies with Fluorophores. Monoclonal Antibody Protocols.

[50] Würth C., Grabolle M., Pauli J., Spieles M., Resch-Genger U. (2013). Relative and Absolute Determination of Fluorescence Quantum Yields of Transparent Samples. Nat. Protoc..

[51] Bannwarth C., Caldeweyher E., Ehlert S., Hansen A., Pracht P., Seibert J., Spicher S., Grimme S. (2021). Extended Tight-Binding Quantum Chemistry Methods. Wiley Interdiscip. Rev. Comput. Mol. Sci..

[52] Pracht P., Bohle F., Grimme S. (2020). Automated Exploration of the Low-Energy Chemical Space with Fast Quantum Chemical Methods. Phys. Chem. Chem. Phys..

[53] Wang J., Durbeej B. (2020). How Accurate are TD-DFT Excited-State Geometries Compared to DFT Ground-State Geometries?. J. Comput. Chem..

[54] Santoro F., Cerezo J., FCclasses3 A Code for Vibronic Calculations. http://www.iccom.cnr.it/en/fcclasses.

[55] Neese F. (2022). Software Update: The ORCA Program System-Version 5.0. Wiley Interdiscip. Rev. Comput. Mol. Sci..

[56] Neese F. (2018). Software Update: The ORCA Program System, Version 4.0. Wiley Interdiscip. Rev. Comput. Mol. Sci..

[57] Neese F. (2012). The ORCA Program System. Wiley Interdiscip. Rev. Comput. Mol. Sci..

[58] Lu T., Chen F. (2012). Multiwfn: A Multifunctional Wavefunction Analyzer. J. Comput. Chem..

[59] Chemcraft-Graphical Software for Visualization of Quantum Chemistry Computations. https://www.chemcraftprog.com.

[60] Humphrey W., Dalke A., Schulten K. (1996). VMD: Visual Molecular Dynamics. J. Mol. Graph..

[61] Morris G.M., Huey R., Lindstrom W., Sanner M.F., Belew R.K., Goodsell D.S., Olson A.J. (2009). AutoDock4 and AutoDockTools4: Automated Docking with Selective Receptor Flexibility. J. Comput. Chem..

[62] Protein Data Bank 4F5S: Crystal Structure of Bovine Serum Albumin. https://www.rcsb.org/structure/4F5S.

[63] Protein Data Bank 7O4X: Crystal Structure of the PII-Like Protein PotN from Lentilactobacillus Hilgardii. https://www.rcsb.org/structure/7O4X.

[64] Morris G.M., Goodsell D.S., Halliday R.S., Huey R., Hart W.E., Belew R.K., Olson A.J. (1998). Automated Docking Using a Lamarckian Genetic Algorithm and an Empirical Binding Free Energy Function. J. Comput. Chem..

[65] Zhu K., Borrelli K.W., Greenwood J.R., Day T., Abel R., Farid R.S., Harder E. (2014). Docking Covalent Inhibitors: A Parameter Free Approach to Pose Prediction and Scoring. J. Chem. Inf. Model..

[66] Pettersen E.F., Goddard T.D., Huang C.C., Couch G.S., Greenblatt D.M., Meng E.C., Ferrin T.E. (2004). UCSF Chimera—A Visualization System for Exploratory Research and Analysis. J. Comput. Chem..

