# Supplementary material for: Amine-Reactive BODIPY Dye: Spectral Properties and Application for Protein Labeling
Source: Molecules. 2022 Nov 16;27(22):7911. doi: 10.3390/molecules27227911 (PMC9692440; doi:10.3390/molecules27227911)
Supplement: Supplementary file 1 [file molecules-27-07911-s001.zip › molecules-2022608-SI.pdf]

Supplementary Materials

Article

Amine-Reactive BODIPY Dye: Spectral Properties and Application for Protein Labeling

Ksenia V. Ksenofontova [1,*], Anastasia A. Kerner [1], Alexander A. Ksenofontov [1,2], Artyom Yu. Shagurin [3], Pavel S. Bocharov [2], Michael M. Lukanov [1,2], Airat R. Kayumov [4], Darya E. Zhuravleva [4], Zalina I. Iskhakova [4], Evgeniy E. Molchanov [5], Dmitriy A. Merkushev [1], Ilya A. Khodov [2] and Yuriy S. Marfin [1,6]

[1] Department of Inorganic Chemistry, Ivanovo State University of Chemistry and Technology, 10, Sheremetevskiy pr., 153000 Ivanovo, Russia
[2] G. A. Krestov Institute of Solution Chemistry of The Russian Academy of Sciences, 1, ul. Akademicheskaya, 153045 Ivanovo, Russia
[3] Laboratoire de Spectroscopie pour les Interactions, la Réactivité et l'Environnement (UMR CNRS 8516), Université de Lille, CEDEX, 59655 Villeneuve d'Ascq, France
[4] Institute of Fundamental Medicine and Biology, Kazan Federal University, 18, ul. Kremlyovskaya, 420008 Kazan, Russia
[5] Department of Fine Organic Synthesis Technology, Ivanovo State University of Chemistry and Technology, 7, Sheremetevskiy pr., 153000 Ivanovo, Russia
[6] Pacific National University, 136, ul. Tihookeanskaya, 680042 Khabarovsk, Russia
* Correspondence: kvk@isuct.ru

Figure S1. Fluorescence decay curves of **COOH-Ph-BODIPY** (blue) and **NHS-Ph-BODIPY** (green) in DMSO (*a*) and *n*-propanol (*b*). 3

S1. Description of Solvent Effects 4

Table S1. Regression coefficients y_0, $a - c$ and coefficients of determination R^2 for multiple linear regression analysis of maximum absorption $v_{abs}(max)$ and emission $v_{em}(max)$ wavenumbers and Stokes shifts Δv of **COOH-Ph-BODIPY** and **NHS-Ph-BODIPY** as a function of Kamlet–Taft solvent scale parameters {α, β, π*} 5

Table S2. Regression coefficients y_0, $a - d$ and coefficients of determination R^2 for multiple linear regression analysis of maximum absorption $v_{abs}(max)$ and emission $v_{em}(max)$ wavenumbers of **COOH-Ph-BODIPY** and **NHS-Ph-BODIPY** as a function of Catalán solvent scale parameters {*SB*, *SP*, *SdP*}, {*SA*, *SP*, *SdP*}, {*SA*, *SB*, *SdP*}, and {*SA*, *SB*, *SP*}. 6

Figure S2. Lippert plots for **COOH-Ph-BODIPY** (*a*) and **NHS-Ph-BODIPY** (*b*) (numbers refer to organic solvents in Table 1). 7

Figure S3. First ten singlet excitation energies and their oscillator strengths *f* for each of two conformers (square – conformer 1, circle – conformer 2) of **COOH-Ph-BODIPY** (blue) and **NHS-Ph-BODIPY** (green) according to TD-DFT calculations. 8

Figure S4. Fluorescence decay curves of **NHS-Ph-BODIPY** (green) and **BSA–NHS-Ph-BODIPY** (orange) in DMSO–bicarbonate buffer (pH 8.3) mixture (1:9). 9

Figure S5. Synchronous emission spectra of BSA (grey) and **BSA–NHS-Ph-BODIPY** (orange) in DMSO–bicarbonate buffer (pH 8.3) mixture (1:9). 10

Figure S6. Amino acid environment of **NHS-Ph-BODIPY** in BSA according to covalent docking results of **Lys187-BSA–NHS-Ph-BODIPY** (*a*), **Lys221-BSA–NHS-Ph-BODIPY** (*b*), and **Lys294-BSA–NHS-Ph-BODIPY** (*c*) systems. 11

Figure S7. Amino acid environment of **NHS-Ph-BODIPY** in PotN according to covalent docking results of **Lys92-PotN – NHS-Ph-BODIPY** system. 12

Figure S8. ^{1}H NMR spectra of **COOH-Ph-BODIPY** (*a*) and **NHS-Ph-BODIPY** (*b*). 13

Figure S9. ^{11}B NMR spectra of **COOH-Ph-BODIPY** (*a*) and **NHS-Ph-BODIPY** (*b*). 14

Figure S10. IR spectra of **COOH-Ph-BODIPY** (*a*) and **NHS-Ph-BODIPY** (*b*). 15

Figure S11. MS spectra of **COOH-Ph-BODIPY** (*a*) and **NHS-Ph-BODIPY** (*b*). 16

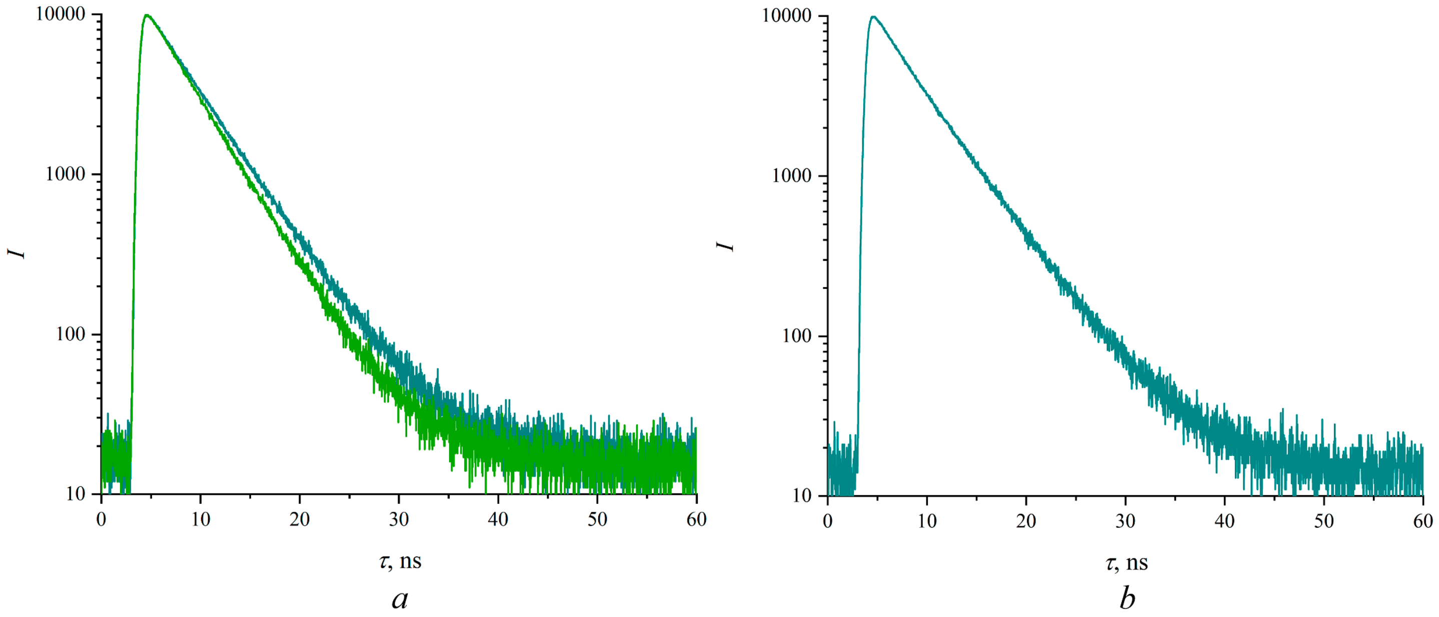

Figure S1. Fluorescence decay curves of **COOH-Ph-BODIPY** (blue) and **NHS-Ph-BODIPY** (green) in DMSO (*a*) and *n*-propanol (*b*).

S1. Description of Solvent Effects

In order to describe solvent effects on spectral characteristics of the compounds investigated, the Lippert–Mataga equation (Equation (S1)) [29] and a multiparameter approach using the Kamlet–Taft equation (Equation (S2)) [27] were applied.

$$\Delta\nu = \frac{2}{hc}\left[\frac{\varepsilon - 1}{2\varepsilon + 1} - \frac{n^2 - 1}{2n^2 + 1}\right]\frac{\left(\mu_{ex} - \mu_{gr}\right)^2}{a^3} \qquad \text{(S1)}$$

where $\Delta\nu$ is the Stokes shift, h is the Planck's constant, c is the speed of a light, ε is the dielectric constant of a solvent, n is the refractive index of a solvent, μ_{ex} and μ_{gr} are the dipole moments of a solute in excited and ground states, respectively, a is the radius of a cavity in which a solute resides. The term in the square brackets is called the orientation polarizability and is denoted as Δf.

$$y = y_0 + a_\alpha\alpha + b_\beta\beta + c_{\pi^*}\pi^* \qquad \text{(S2)}$$

where y is the value of a solvent-dependent physicochemical property in a given solvent, y_0 is the statistical quantity corresponding to the value of a solvent-dependent physicochemical property in the gas phase or in an inert solvent, α is the empirical parameter of solvent hydrogen-bond donor acidity, β is the empirical parameter of solvent hydrogen-bond acceptor basicity, π^* is the empirical parameter of solvent dipolarity/polarizability, a – c are the regression coefficients describing the sensitivity of a physicochemical property y to the different solute/solvent interaction mechanisms.

Table S1. Regression coefficients y_0, $a - c$ and coefficients of determination R^2 for multiple linear regression analysis of maximum absorption $\nu_{abs}(max)$ and emission $\nu_{em}(max)$ wavenumbers and Stokes shifts $\Delta\nu$ of **COOH-Ph-BODIPY** and **NHS-Ph-BODIPY** as a function of Kamlet–Taft solvent scale parameters $\{\alpha, \beta, \pi^*\}$.

	y_0	a_α	b_β	c_{π^*}	R^2
**COOH-Ph-BODIPY**					
$\nu_{abs}(max)$, cm^{-1}	18,982±64	−49±113	295±129	−89±114	0.56
$\nu_{em}(max)$, cm^{-1}	18,377±69	−36±122	372±139	−152±123	0.66
$\Delta\nu$, cm^{-1}	605±37	−13±66	−77±75	64±66	0.31
**NHS-Ph-BODIPY**					
$\nu_{abs}(max)$, cm^{-1}	18,913±56	−60±100	222±114	−108±100	0.44
$\nu_{em}(max)$, cm^{-1}	18,292±57	−105±101	296±115	−195±102	0.57
$\Delta\nu$, cm^{-1}	621±17	45±30	−74±34	87±30	0.61

Table S2. Regression coefficients y_0, $a - d$ and coefficients of determination R^2 for multiple linear regression analysis of maximum absorption $\nu_{abs}(max)$ and emission $\nu_{em}(max)$ wavenumbers of **COOH-Ph-BODIPY** and **NHS-Ph-BODIPY** as a function of Catalán solvent scale parameters {*SB*, *SP*, *SdP*}, {*SA*, *SP*, *SdP*}, {*SA*, *SB*, *SdP*}, and {*SA*, *SB*, *SP*}.

	y_0	a_{SA}	b_{SB}	c_{SP}	d_{SdP}	R^2
**COOH-Ph-BODIPY**						
$\nu_{abs}(max)$, cm^{-1}	19,538±127	–	86±61	−963±187	210±53	0.91
	19,602±145	9±103	–	−1,055±214	258±50	0.89
	18,900±67	−28±258	213±173	–	93±115	0.54
	19,434±223	−165±199	301±110	−707±312	–	0.72
$\nu_{em}(max)$, cm^{-1}	18,902±263	–	190±125	−927±387	156±110	0.75
	18,969±287	178±204	–	−1,018±424	234±100	0.69
	18,290±83	89±319	261±214	–	54±142	0.52
	18,809±297	−30±265	315±147	−707±415	–	0.67
**NHS-Ph-BODIPY**						
$\nu_{abs}(max)$, cm^{-1}	19,437±103	–	31±49	−901±152	160±43	0.91
	19,499±95	−78±68	–	−991±140	191±33	0.92
	18,840±61	−90±233	178±156	–	44±104	0.38
	19,374±156	−208±139	225±77	−733±218	–	0.78
$\nu_{em}(max)$, cm^{-1}	18,875±152	–	97±73	−1,004±224	98±64	0.85
	18,988±165	−76±118	–	−1,168±244	166±57	0.82
	18,209±70	−171±268	292±180	–	−38±119	0.39
	18,865±120	−280±107	271±59	−952±168	–	0.90

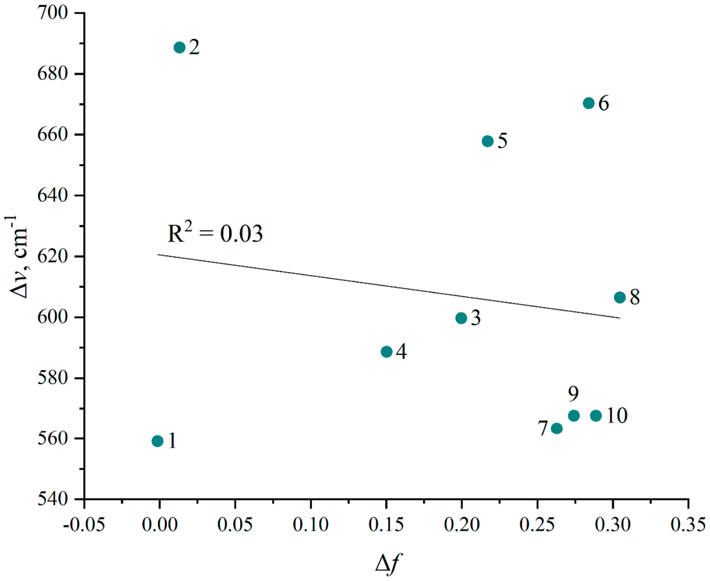

a

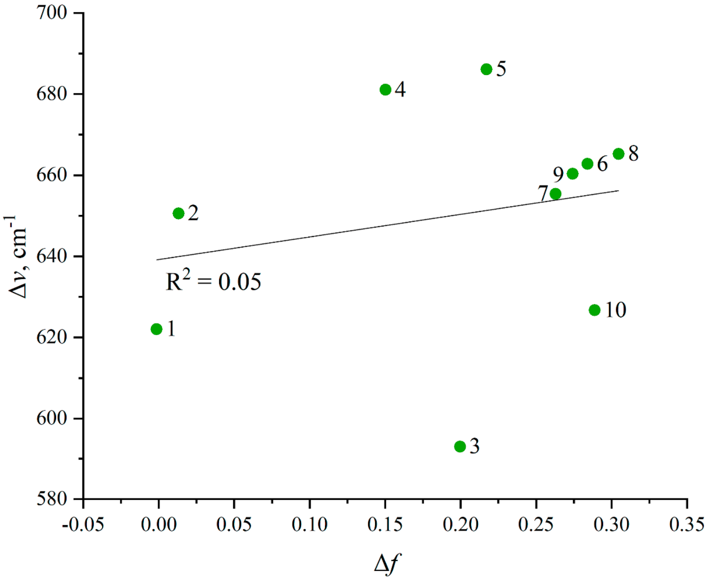

b

Figure S2. Lippert plots for **COOH-Ph-BODIPY** (*a*) and **NHS-Ph-BODIPY** (*b*) (numbers refer to organic solvents in Table 1).

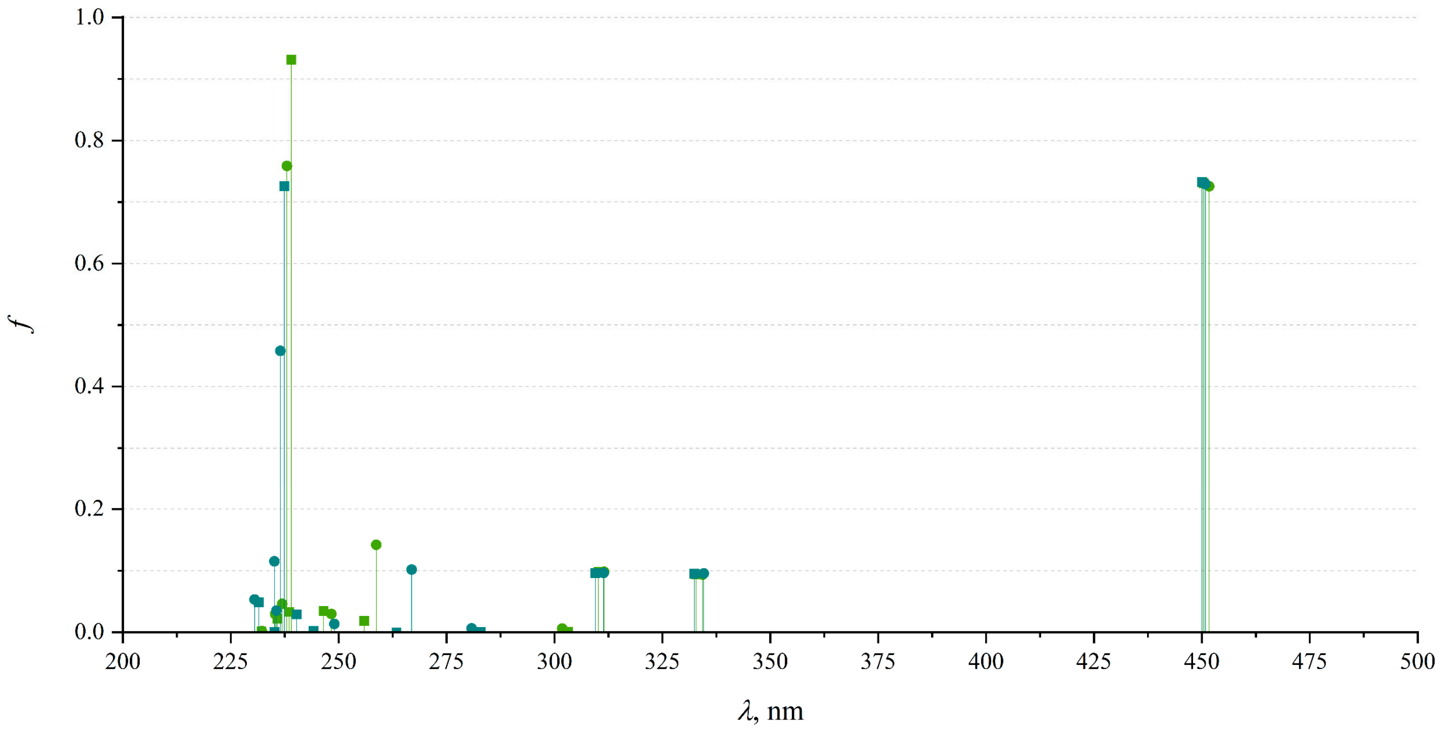

Figure S3. First ten singlet excitation energies and their oscillator strengths *f* for each of two conformers (square – conformer 1, circle – conformer 2) of **COOH-Ph-BODIPY** (blue) and **NHS-Ph-BODIPY** (green) according to TD-DFT calculations.

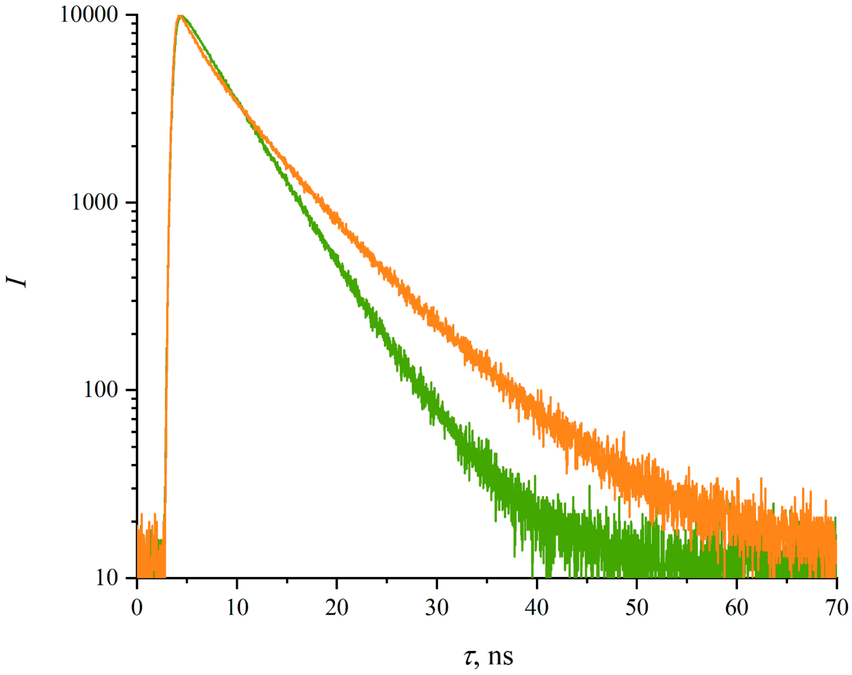

Figure S4. Fluorescence decay curves of **NHS-Ph-BODIPY** (green) and **BSA–NHS-Ph-BODIPY** (orange) in DMSO–bicarbonate buffer (pH 8.3) mixture (1:9).

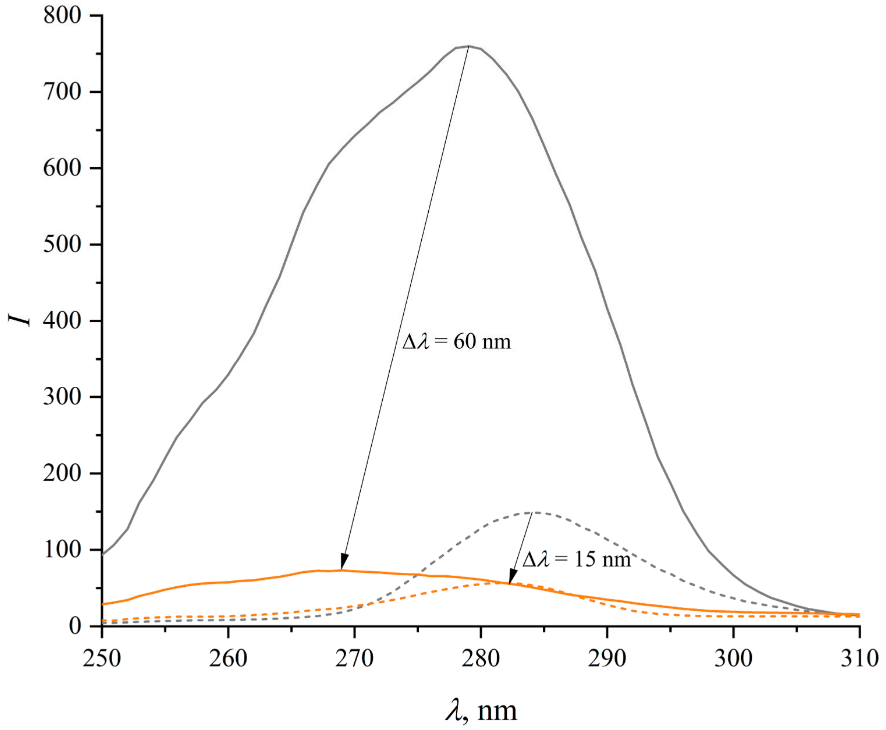

Figure S5. Synchronous emission spectra of BSA (grey) and **BSA–NHS-Ph-BODIPY** (orange) in DMSO–bicarbonate buffer (pH 8.3) mixture (1:9).

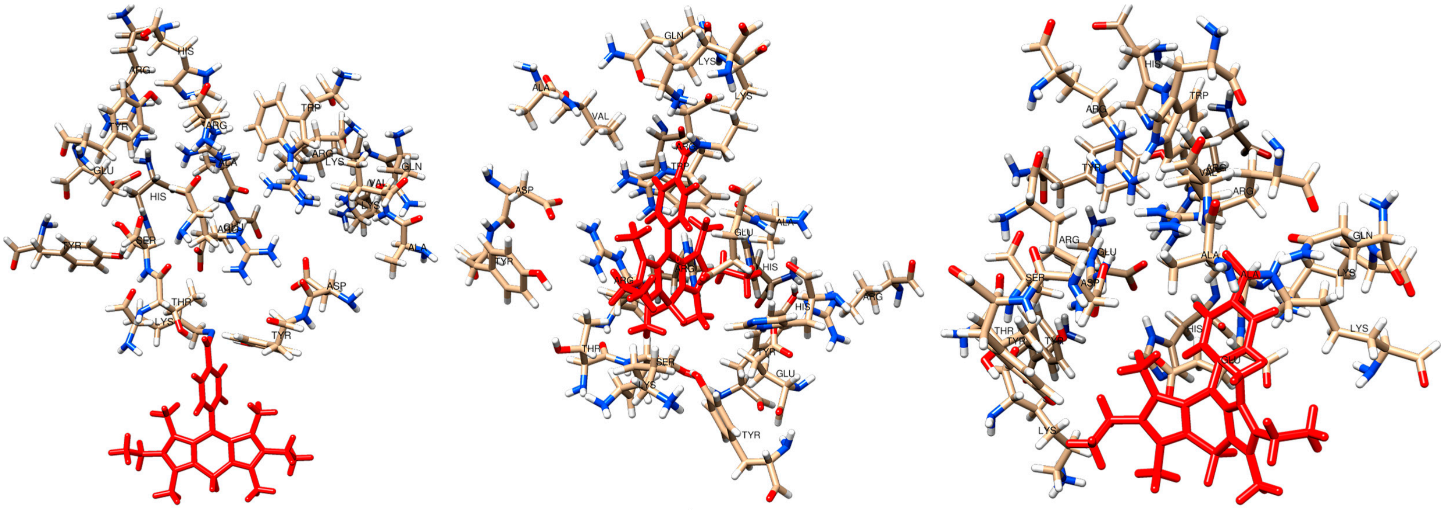

a *b* *c*

Figure S6. Amino acid environment of **NHS-Ph-BODIPY** in BSA according to covalent docking results of **Lys187-BSA–NHS-Ph-BODIPY** (*a*), **Lys221-BSA–NHS-Ph-BODIPY** (*b*), and **Lys294-BSA–NHS-Ph-BODIPY** (*c*) systems.

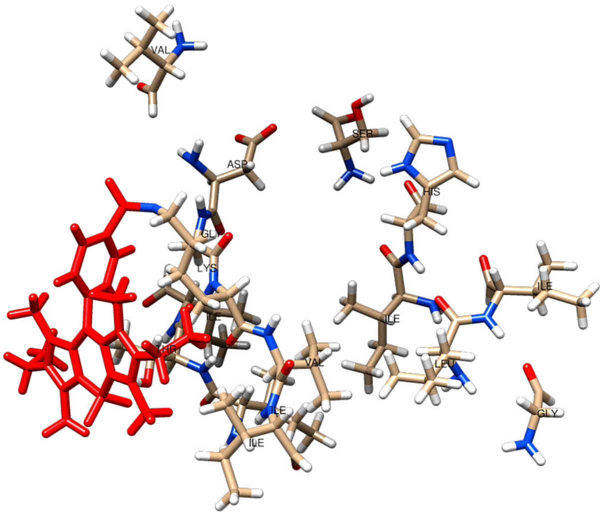

Figure S7. Amino acid environment of **NHS-Ph-BODIPY** in PotN according to covalent docking results of **Lys92-PotN – NHS-Ph-BODIPY** system.

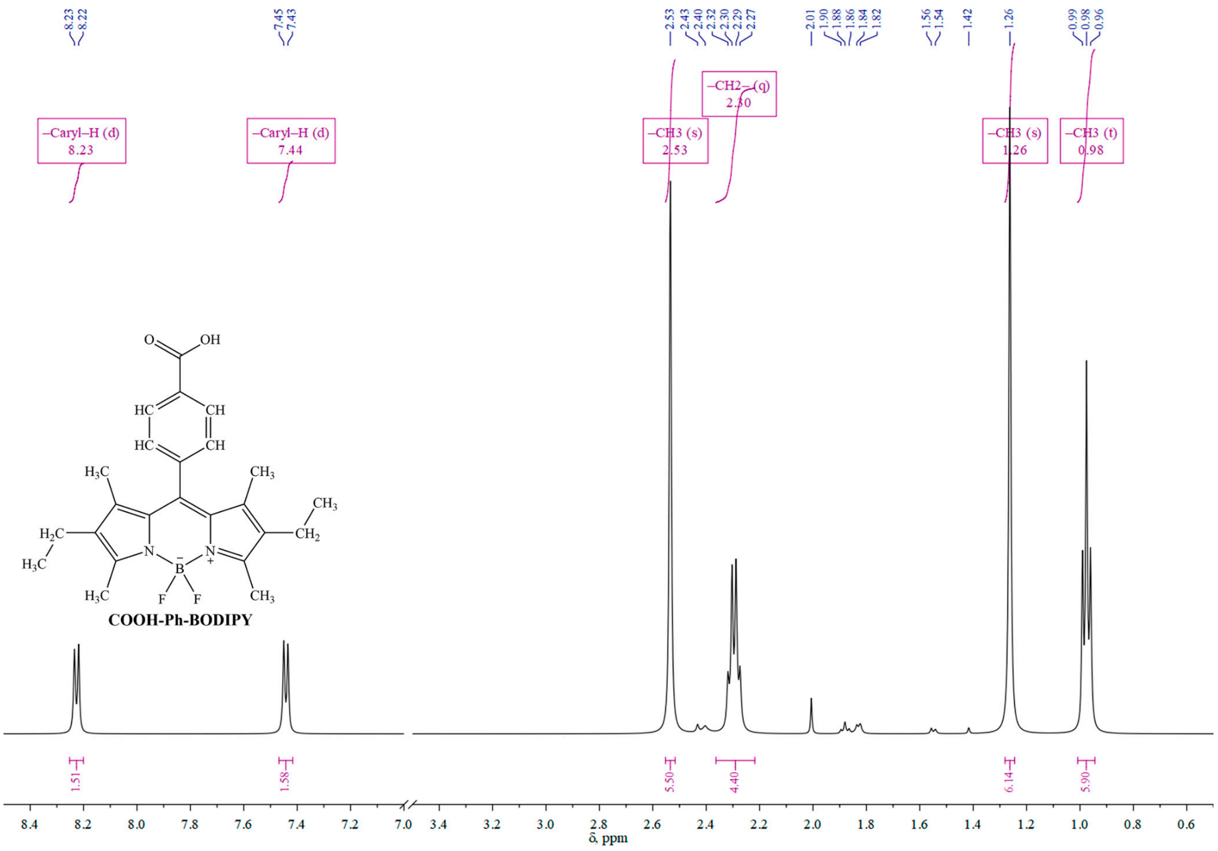

a

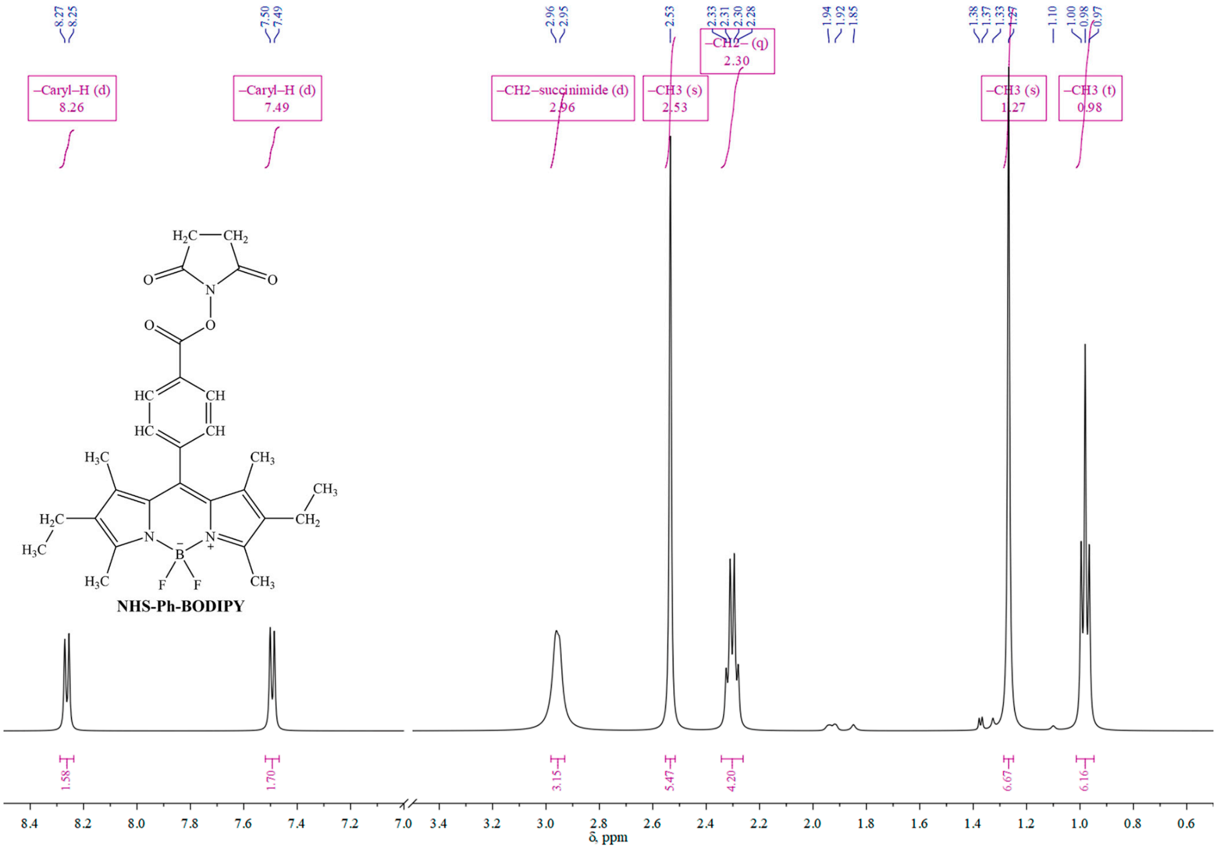

b

Figure S8. ^{1}H NMR spectra of **COOH-Ph-BODIPY** (*a*) and **NHS-Ph-BODIPY** (*b*).

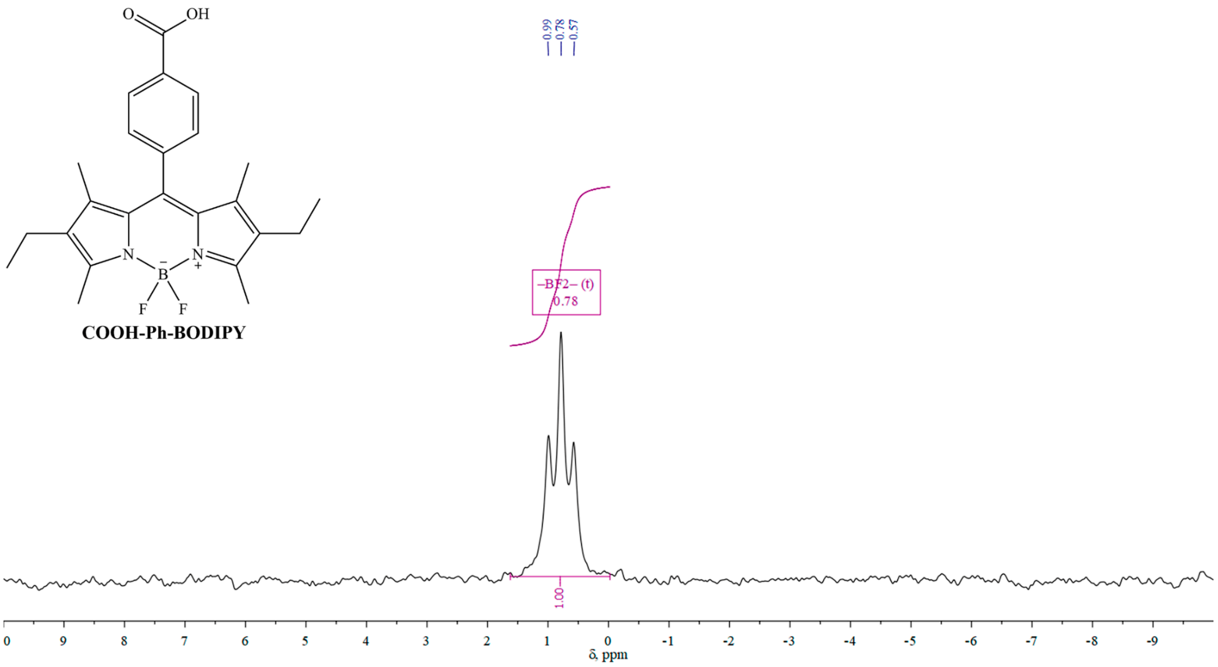

a

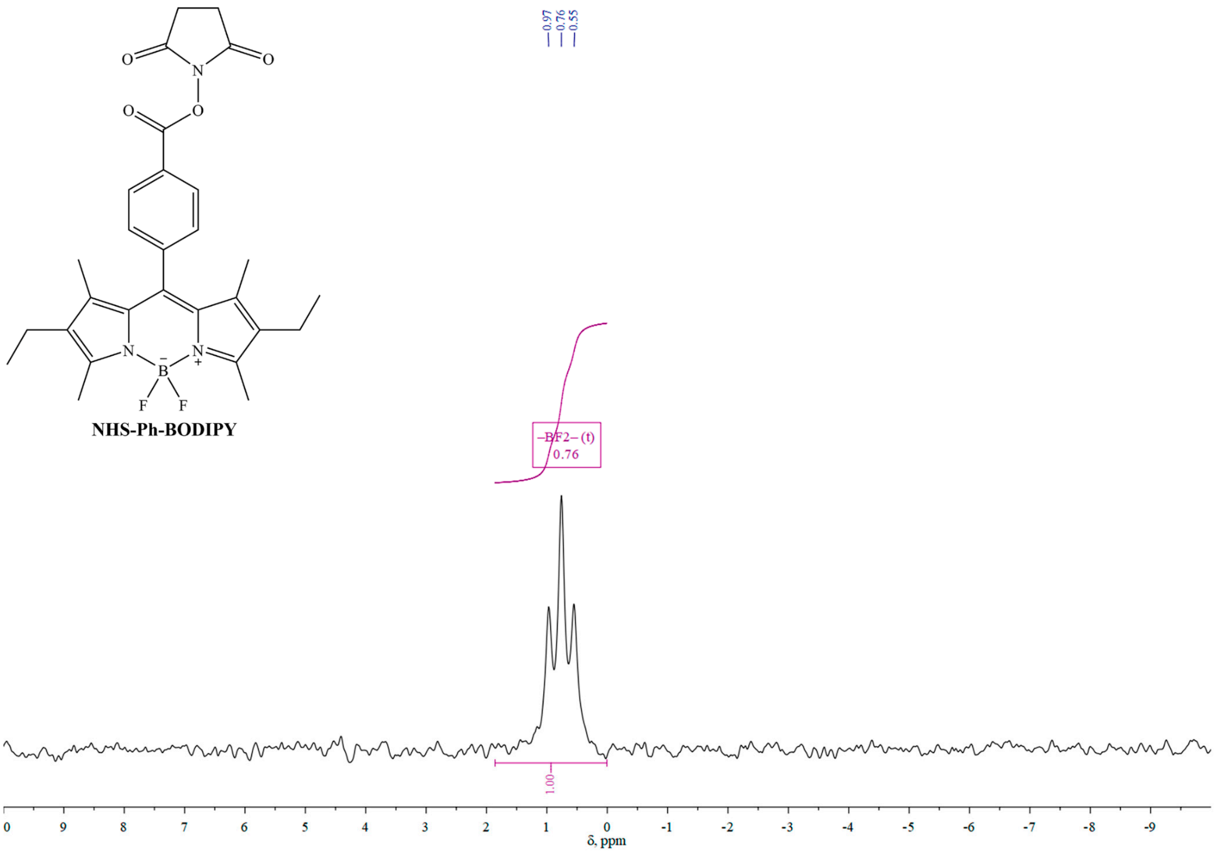

b

Figure S9. ^{11}B NMR spectra of **COOH-Ph-BODIPY** (*a*) and **NHS-Ph-BODIPY** (*b*).

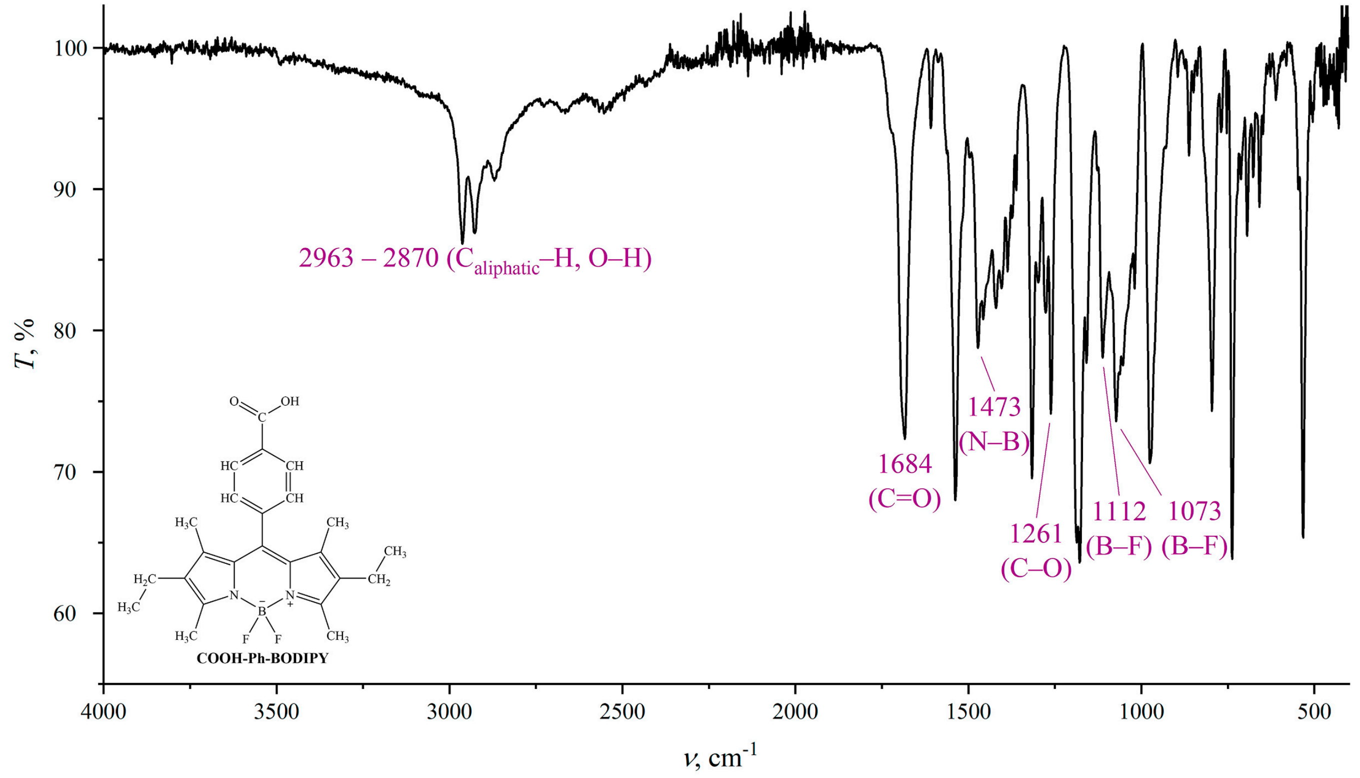

a

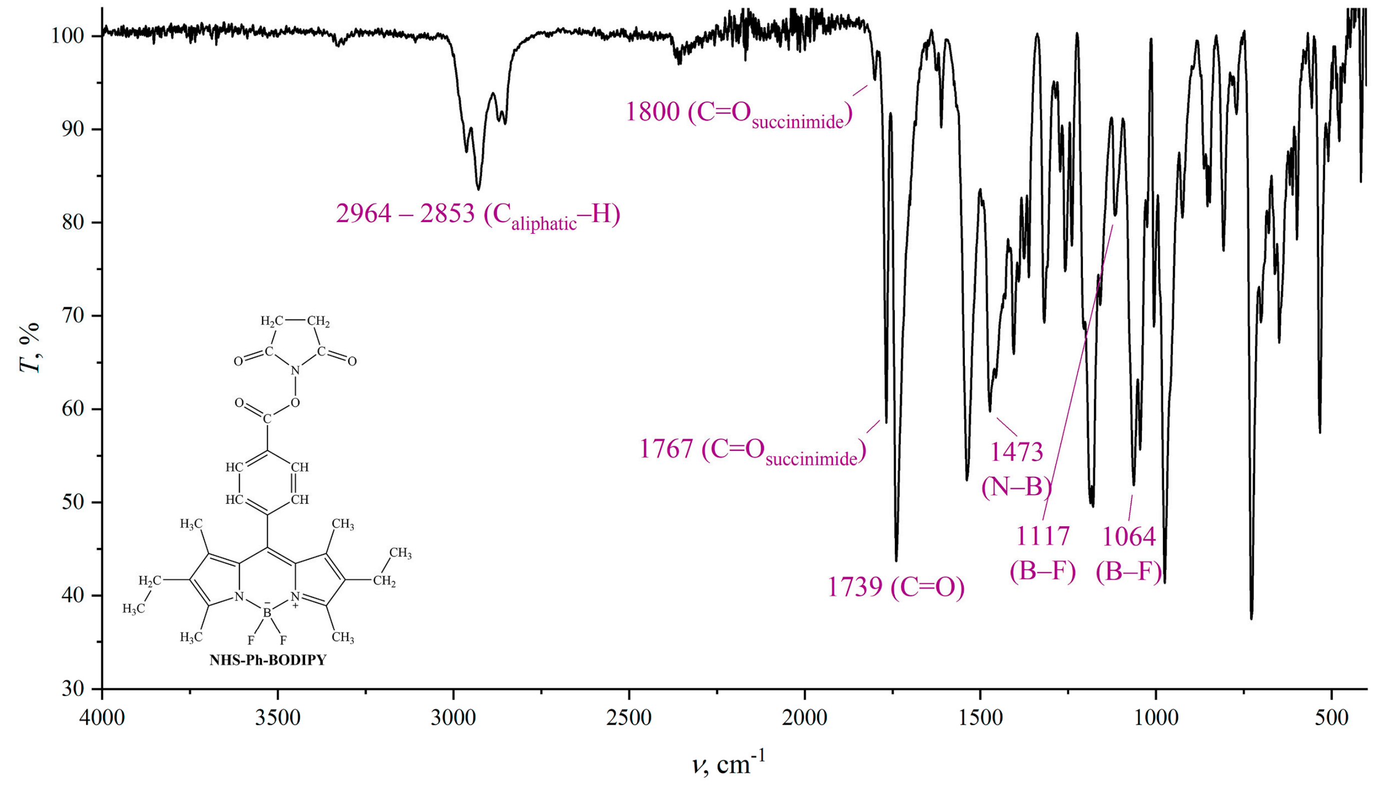

b

Figure S10. IR spectra of **COOH-Ph-BODIPY** (*a*) and **NHS-Ph-BODIPY** (*b*).

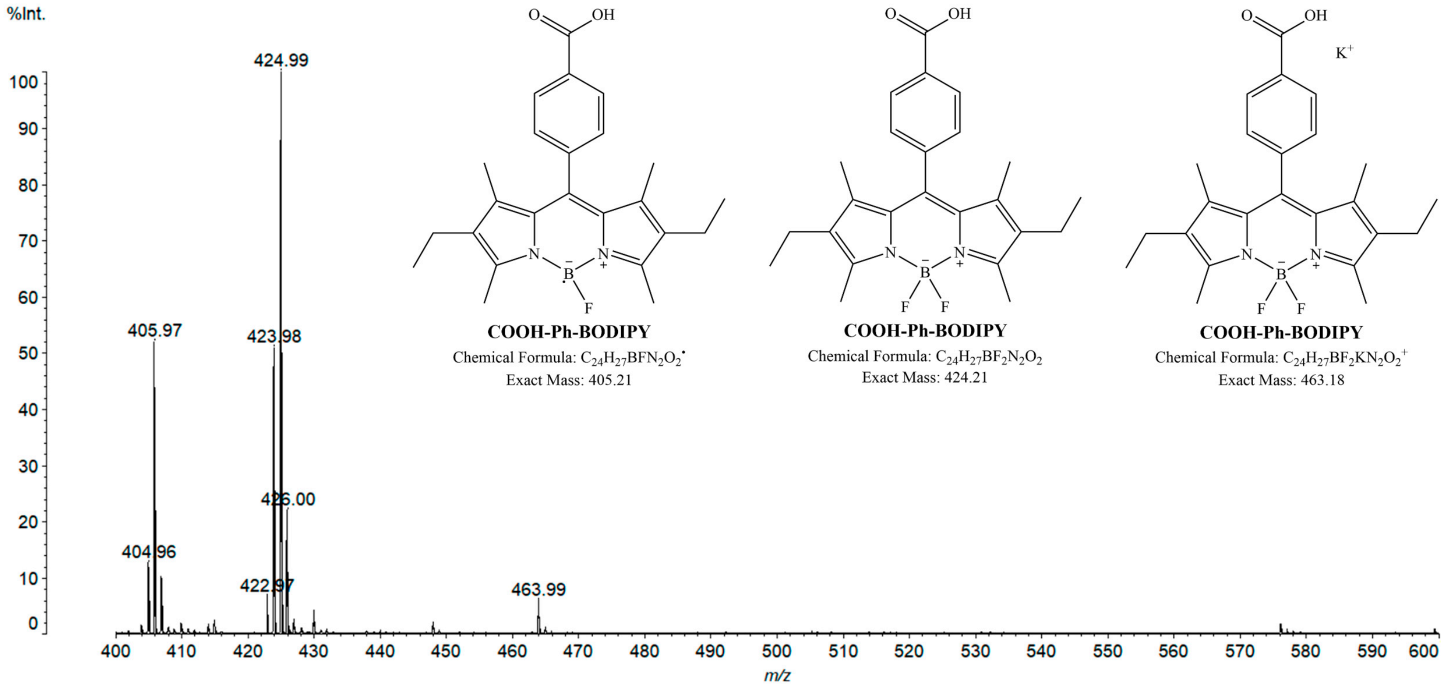

a

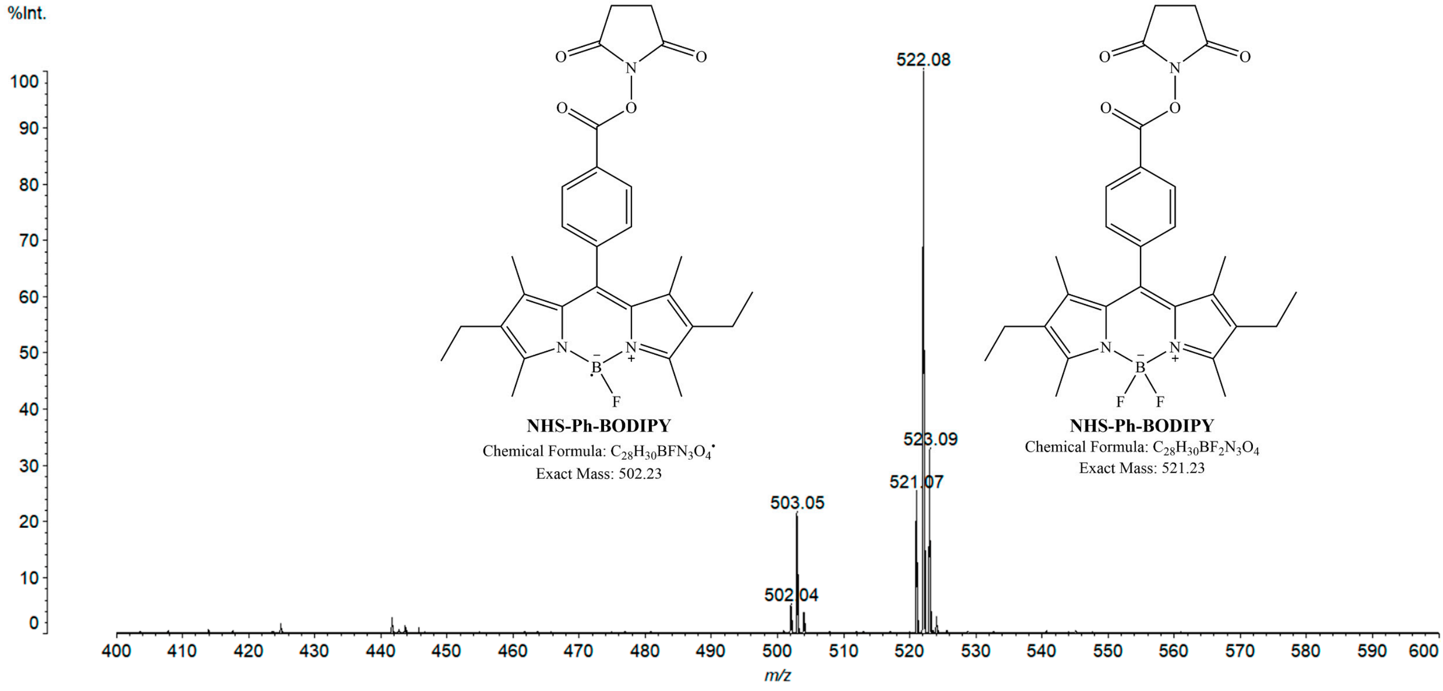

b

Figure S11. MS spectra of **COOH-Ph-BODIPY** (*a*) and **NHS-Ph-BODIPY** (*b*).
